# Comparative transcriptional profiling of *Gracilariopsis lemaneiformis* in response to salicylic acid- and methyl jasmonate-mediated heat resistance

**DOI:** 10.1371/journal.pone.0176531

**Published:** 2017-05-02

**Authors:** Fangjun Wang, Chongbin Wang, Tonglei Zou, Nianjun Xu, Xue Sun

**Affiliations:** 1 School of Marine Sciences, Ningbo University, Ningbo, Zhejiang, China; 2 Key Laboratory of Marine Biotechnology of Zhejiang Province, Ningbo, China; Agriculture and Agri-Food Canada, CANADA

## Abstract

Culturing the economically important macroalga *Gracilariopsis lemaneiformis* (Rhodophyta) is limited due to the high temperatures in the summertime on the southern Chinese coast. Previous studies have demonstrated that two phytohormones, salicylic acid (SA) and methyl jasmonate (MJ), can alleviate the adverse effects of high-temperature stress on *Gp*. *lemaneiformis*. To elucidate the molecular mechanisms underlying SA- and MJ-mediated heat tolerance, we performed comprehensive analyses of transcriptome-wide gene expression profiles using RNA sequencing (RNA-seq) technology. A total of 14,644 unigenes were assembled, and 10,501 unigenes (71.71%) were annotated to the reference databases. In the SA, MJ and SA/MJ treatment groups, 519, 830, and 974 differentially expressed unigenes were detected, respectively. Unigenes related to photosynthesis and glycometabolism were enriched by SA, while unigenes associated with glycometabolism, protein synthesis, heat shock and signal transduction were increased by MJ. A crosstalk analysis revealed that 216 genes were synergistically regulated, while 18 genes were antagonistically regulated by SA and MJ. The results indicated that the two phytohormones could mitigate the adverse effects of heat on multiple pathways, and they predominantly acted synergistically to resist heat stress. These results will provide new insights into how SA and MJ modulate the molecular mechanisms that counteract heat stress in algae.

## Introduction

*Gracilariopsis lemaneiformis* (Rhodophyta) is regarded as one of the most important commercial species of algae due to its use in high-quality agar production. Additionally, the large-scale cultivation of *Gp*. *lemaneiformis* can be an effective bioremediation measure for eutrophication control in seawater [1]. Wild *Gp*. *lemaneiformis* originated on the northern coast of China, and heat-resistant strain 981 of this alga was extensively cultivated on China’s southern coasts. However, high temperatures in the summertime have limited the *Gp*. *lemaneiformis* industry. High temperatures can reduce the algal growth rate, shorten its cultivation period, and ultimately decrease the algal yield.

High temperatures can adversely affect photosynthesis, respiration, water balance and membrane stability. They also modulate hormone levels, primary and secondary metabolites [2]. Recently, numerous reports have demonstrated that phytohormones play roles in plant growth and development as well as biotic and abiotic stresses. Salicylic acid (SA), a small phenolic phytohormone compound, has been shown to play a role in heat resistance [3]. SA enhances early-stage H_2_O_2_ levels during heat stress [4] and alleviates heat-induced membrane injuries. It also significantly enhances proline, ascorbic acid, and glutathione levels with concomitant inductions of various stress-related enzymes to resist heat stress [5,6]. Moreover, SA maintains Ca^2+^ homeostasis and alleviates decreases in the net photosynthesis rate during heat stress [6,7].

Jasmonates (JAs), such as jasmonic acid (JA) and methyl jasmonate (MeJA or MJ), are a ubiquitous group of lipid-based phytohormones that are derived from α-linolenic acid. JAs serve as endogenous signaling molecules in plant growth and development. They also have potential functions in biotic and abiotic defenses [8]. A previous study demonstrated that the effect of MJ on heat tolerance was stronger than that of SA in *Phalaenopsis* seedlings [9]. Similar to SA, MJ increases reactive oxygen species (ROS) levels, the photochemical efficiency of photosystem II (*F*_v_/*F*_m_) and the actual photochemical efficiency (*Φ*_PSII_) at high temperatures [10,11].

SA and MJ display similar effects in improving stress tolerance in higher plants, but the interactions between SA and JAs are complex. For example, patterns of one-way, mutual antagonistic and synergistic effects on the regulation of several genes have been observed in *Sorghum bicolor* by microarray analysis [12]. Although few studies have addressed crosstalk between the SA and MJ pathways during high-temperature stress, JAs are known to cooperate with SA to confer a basal thermotolerance [13].

Our previous studies demonstrated that SA and MJ enhanced the growth rate, proline levels and various physiological and biochemical processes in *Gp*. *lemaneiformis* under heat stress [14]. In this study, RNA sequencing (RNA-seq) and gene expression profiling analyses were performed in *Gp*. *lemaneiformis* to compare SA and MJ effects under high-temperature conditions. The aim of this study was to provide data that would contribute to a comprehensive understanding of the metabolic changes and signaling pathways mediated by SA and MJ against heat stress. This work will shed light on the role of phytohormones in algae.

## Materials and methods

### Algal materials and culture conditions

The alga *Gp*. *lemaneiformis* 981 was collected from the coast of Ningde (26°65´N, 119°66´E), Fujian Province, China, in September 2009. *Gp*. *lemaneiformis* is a kind of common macroalga, which was cultured in large areas in Southeastern Coast of China, so we were permitted to collect a small quantity sample from one farmer. And this study did not involve any endangered or protected species. The tender thalli were repeatedly cut off and thoroughly washed in sterile seawater to remove sediment and epiphytes. The purified algae were cultured in Provasoli medium at 23°C under a 12 L: 12 D photoperiod with an approximately 40 μmol photons·m^-2^·s^-1^ light intensity. Approximately 600 mg algal apexes (3–4 cm) were cultivated in a 1L flask with fresh medium added every three days and were then used for the experiments after one week.

### Phytohormone treatments

Our previous study indicated that 100 μmol/L SA (SA100) and 50 μmol/L MJ (MJ50) had maximal growth effects on *Gp*. *lemaneiformis* at 33°C. Therefore, we established SA100, MJ50, SA100/MJ50, and one control group (CK) for the experiments below. The algal samples for each treatment group were cultured at 33°C for 6 h, and approximately 200 mg of the thalli was used for each RNA extraction.

### RNA isolation

Each sample was ground into a fine powder in liquid nitrogen, and the total RNA was extracted using the RNeasy Plant Mini Kit (Qiagen, Germany). The residual DNA was removed with RNase-free DNase I according to the manufacturer’s instructions. The RNA qualities were monitored on 1% agarose gels. The RNA quantities and purities were assessed using a NanoDrop 1000 Spectrophotometer (Thermo Scientific, Massachusetts, USA).

### cDNA library construction and RNA-seq

Qualified RNAs were used to construct cDNA libraries for each of the four treatment groups. The cDNA libraries were constructed according to the manufacturer’s instructions (Illumina). Library quality was assessed using an Agilent 2100 Bioanalyzer and an ABI StepOnePlus Real-Time PCR System. The cDNA libraries were sequenced on an Illumina HiSeq 2000 using the paired-end technology in a single run. The sequencing reads were deposited in the National Center for Biotechnology (NCBI) Short Read Archive (SRA) database (http://www.ncbi.nlm.nih.gov/sra/) under the accession number SRX2508635 for the control group (CK) and SRX2508642-SRX2508644 for the SA, MJ, and SA/MJ groups, respectively.

### Filtering of reads and *de novo* assembly

The raw data (raw reads) in FASTQ format were filtered. Together with the low-quality reads (those exceeding 20% low-quality bases with Q≤10), reads that contained adaptor sequences or unidentified nucleotides in excess of 5% were removed from the total read pool to obtain clean reads. *De novo* assembly of the clean reads was performed using the Trinity program. After gene family clustering, the final unigenes comprised two classes: clusters and singletons.

### Unigene annotations and functional classifications

All assembled unigenes were searched against the NR (non-redundant protein) and Swissprot databases to identify their putative mRNA functions using the BLASTx algorithm with an E-value cut-off of 10^−5^. BLASTn was also used to align unique sequences to the NT (non-redundant nucleotide) database with the same E-value. ESTscan software was used for the unaligned unigenes, which were translated into peptide sequences (http://www.ch.embnet.org/software/ESTScan2.html). The Gene Ontology (GO) annotations of the unigenes were performed using the BLAST2GO program (http://www.blast2go.com/b2ghome), and WEGO software (http://wego.genomics.org.cn/cgi-bin/wego/index.pl) was used for the GO functional classifications of the unigenes. Kyoto Encyclopedia of Genes and Genomes (KEGG) annotations were performed to identify the metabolic pathway of each unigene using the KEGG Automatic Annotation Server (KAAS) system (http://www.genome.jp/kegg/kaas/).

### Differentially expressed gene (DEG) analysis

Differential expression analysis was performed using the DESeq packages for pairwise comparisons between samples. The transcript abundance of each gene was calculated based on the ratio of the fragments per kb per million fragments (FPKM) values. The resulting *P*-values were adjusted using the Benjamini and Hochberg approach for controlling the false discovery rate (FDR). A gene with a log_2_|fold change|>1 with a FDR significance score <0.001 was defined as a DEG. The GO and KEGG enrichment analyses were performed on the DEGs using the aforementioned method. The GO terms and KEGG pathways with *P*-values <0.05 were considered significantly enriched.

### DEG validation by qPCR

The culture and phytohormone treatment conditions were identical to those used by RNA-seq. The four algal samples were harvested at 6 h to extract the total RNA and to synthesize cDNA using a PrimeScript RT reagent kit with a gDNA Eraser (TaKaRa, Dalian); the cDNA was used as the qPCR template. The expression levels of 30 candidate genes were analyzed by qPCR using an Eppendorf PCR machine (Mastercycler ep realplex, Germany). Each qPCR reaction was represented by six biological replicates. All primers were designed using the Primer Premier 5.0 software and were listed in the S1 Table. The 18S rRNA gene was used as the internal control. The relative transcript abundance was calculated using the 2^-ΔΔCT^ method [15]. The results represent the mean of six replications. A variance analysis of mean values was performed using Duncan’s multiple comparison test with SPSS 13.0 software (SPSS, Chicago, USA), and significant differences were determined at *P*<0.05.

## Results

### Unigene assembly

The number of total raw reads from 4 samples ranged from 57.06 to 58.88 million (M). After the quality filtering, the clean reads varied from 52.09 to 54.55 M. The total lengths of the 4 groups exceeded 4.69 and were lower than 4.91 Gb. The Q20 percentage (the sequencing error rate that was lower than 1%) ranged from 97.87% to 98.30% of the reads. However, the N (ambiguous base) percentage was 0. The GC content was approximately 51.00% for the four groups (Table 1).

**Table 1:** Statistical results from the sequencing data.

Samples	Total+ raw reads	Total clean reads	Total clean nucleotides (nt)	Q20 percent (%)	N percent (%)	GC percent (%)
CK	58,554,626	54,554,420	4,909,897,800	98.20	0.00	51.09
SA	58,881,522	52,087,848	4,687,906,320	97.87	0.00	50.96
MJ	57,570,626	52,998,652	4,769,878,680	98.27	0.00	50.95
SA/MJ	57,059,920	52,422,890	4,718,060,100	98.30	0.00	50.98

After assembly and annotation, 14,644 unigenes with an average length of 2,811 bp and an N50 length of 5,101 bp were obtained. Overall, the consensus unigenes included 5,581 clusters and 9,063 singletons. The lengths of the unigenes all exceeded 200 bp. Unigenes with lengths greater than 3,000 bp (5,227 unigenes) were overrepresented and accounted for approximately 35.69%. Those with lengths of 200–300 bp (2,026 unigenes, 13.84%) were the second most abundant unigene group (S1 Fig).

### Unigene functions and annotated pathways

To characterize the potential functions of the unigenes, we analyzed the 14,644 assembled unigenes against the NR, NT, Swissprot, KEGG, Clusters of Orthologous Groups (COG) and GO databases using the BLAST algorithm with a cut-off E-value of 10^−5^. Ultimately, 10,501 unigenes (71.71%) were annotated in the aforementioned databases (Table 2).

**Table 2:** Summary of unigene annotations in the public databases.

Annotated database	Number of annotated unigenes	Percent of annotated unigenes (%)
NR	10,278	70.19
NT	3,066	20.94
Swissprot	7,568	51.68
KEGG	7,721	52.72
COG	7,074	48.31
GO	3,899	26.63
Annotated unigenes in at least one database	10,501	71.71
Total unigenes	14,644	100

**Fig 1:**
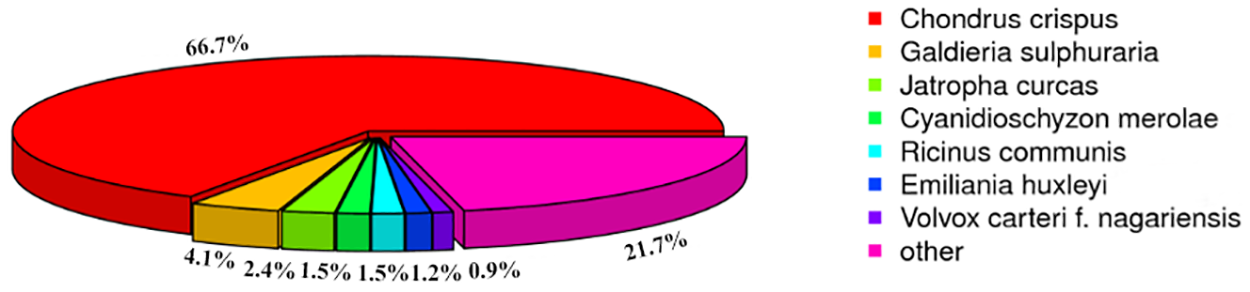
Species distribution of the unigenes against the NR database. The percentages in the pie chart represent the *Gp*. *lemaneiformis* genes that are homologous to those of other species.

Among the annotated reads, 10,278 (70.19%) and 3,066 (20.94%) unigenes showed identities with sequences that had been deposited in the NR and NT databases, respectively. The NR database revealed that the *Gp*. *lemaneiformis* unigenes showed greater similarities to those of *Chondrus crispus* (66.7%) than to those of other species (4.1% match to *Galdieria sulphuraria*, 2.4% match to *Jatropha curcas*, and 21.7% match to 174 species with similarities below 0.9%) (Fig 1). Moreover, 7,568 unigenes (51.68%) showed similarities to proteins in the Swissprot database.

#### GO categories

**Fig 2:**
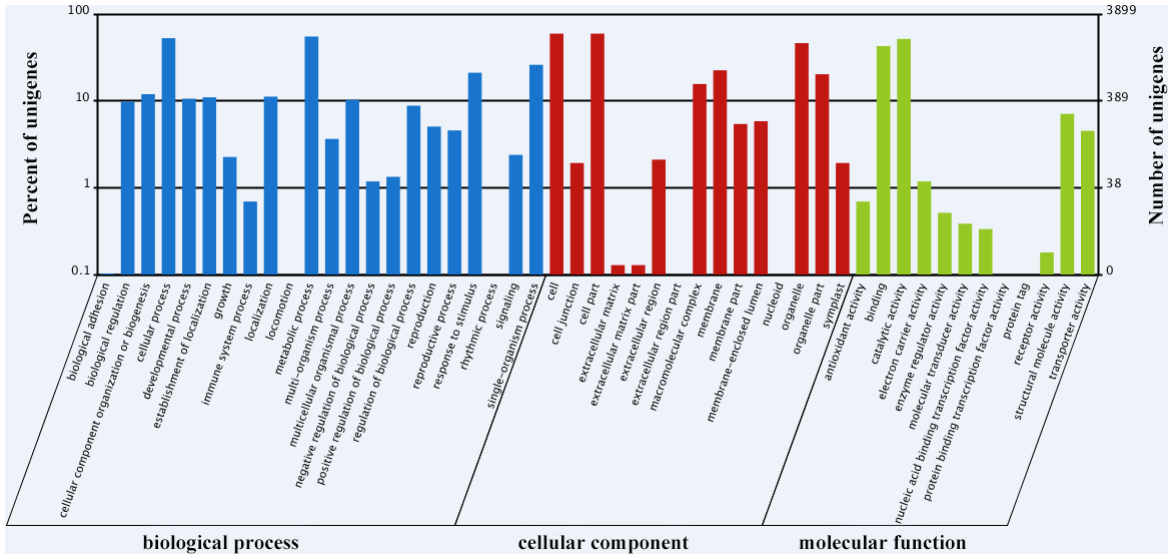
GO categories of the unigenes. The unigenes were annotated to three categories: biological process, cellular component, and molecular function. The abscissa represents the GO category. The left and right ordinates represent percentage and number of the unigenes, respectively.

To evaluate unigene functions, we categorized 3,899 unigenes (26.63%) using GO classification. In the “biological process” category, “metabolic process” was the most dominant group (2,147 unigenes), followed by “cellular process” (2,054 unigenes). In the “cellular component” category, “cell” and “cell part” were the largest GO terms; each consisted of 2,318 unigenes. In the “molecular function” category, most of the corresponding genes were assigned to “catalytic activity” (2,014 unigenes) and “binding” (1,667 unigenes) (Fig 2).

#### COG annotation

**Fig 3:**
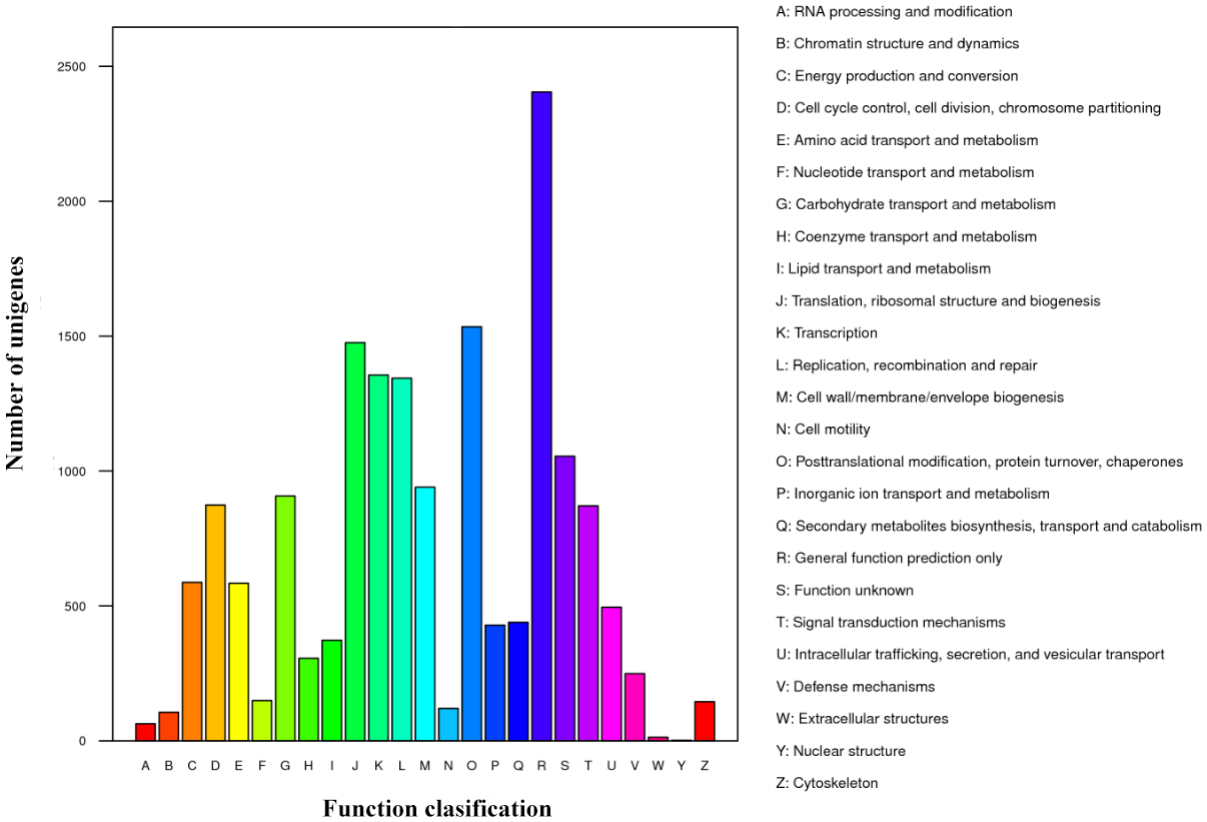
COG functional classification of the unigenes.

Based on the COG analysis, 7,074 (48.31%) non-redundant unigenes were subdivided into 25 clusters (Fig 3). The top COG category was “General function prediction only” (R, 2,405 unigenes), followed by “Posttranslational modification, protein turnover, chaperones” (O, 1,535 unigenes). “Extracellular structures” (W, 13 unigenes) and “Nuclear structure” (Y, 2 unigenes) were poorly characterized.

#### KEGG pathway annotation

**Fig 4:**
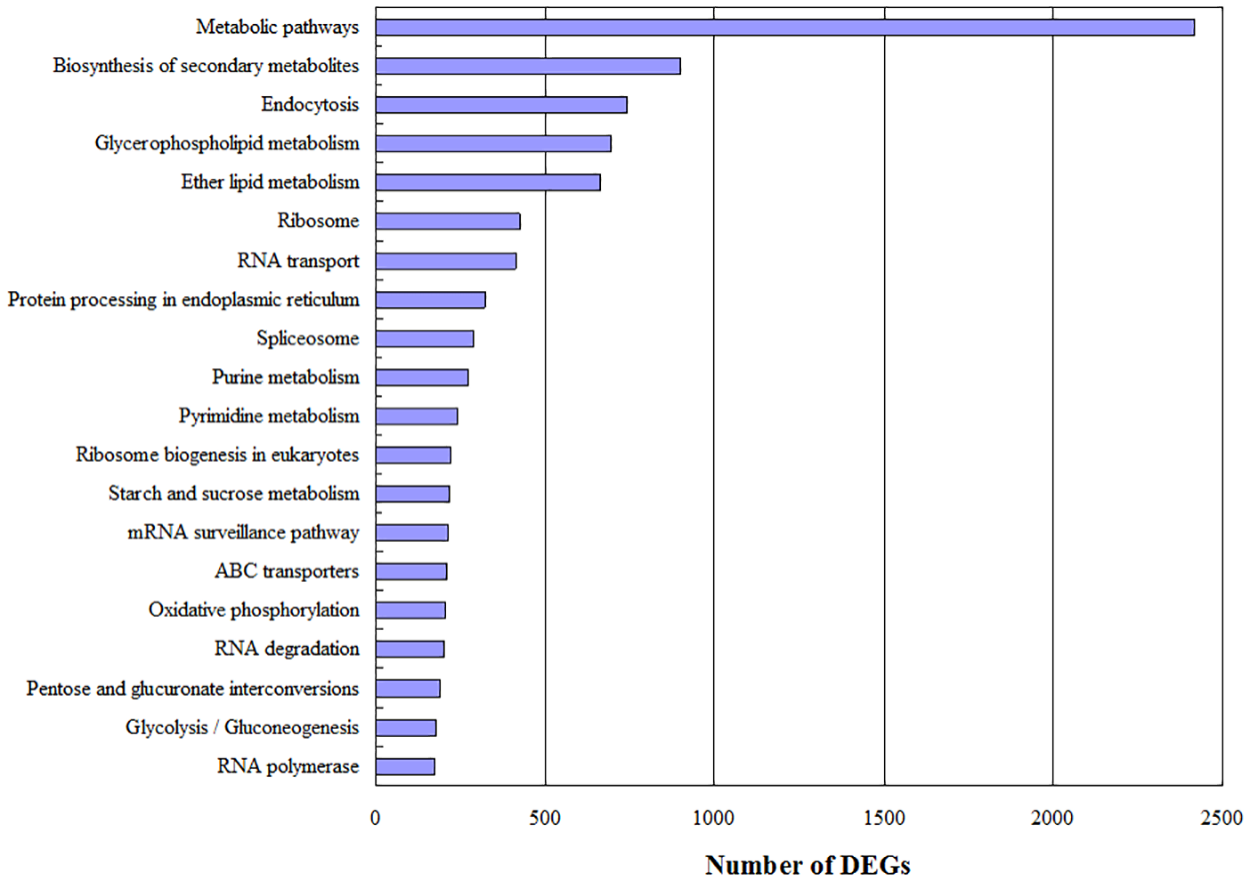
Distribution of pathway numbers against the KEGG database. The top 20 most abundant KEGG pathways were shown.

In the KEGG pathway analysis, 7,721 unigenes (52.72%) were matched in the database and assigned to 125 KEGG pathways. The highly represented pathways included “Metabolism” (2,416 unigenes), “Biosynthesis of secondary metabolites” (899 unigenes), and “Endocytosis” (739 unigenes), “Glycerophospholipid metabolism” (695 unigenes), and “Ether lipid metabolism” (663 unigenes) (Fig 4). The categories with the fewest unigenes included “Betalain biosynthesis”, “Monoterpenoid biosynthesis”, and “Flavone and flavonol biosynthesis”; each contained one unigene.

### DEG profiles in response to the two phytohormones

**Fig 5:**
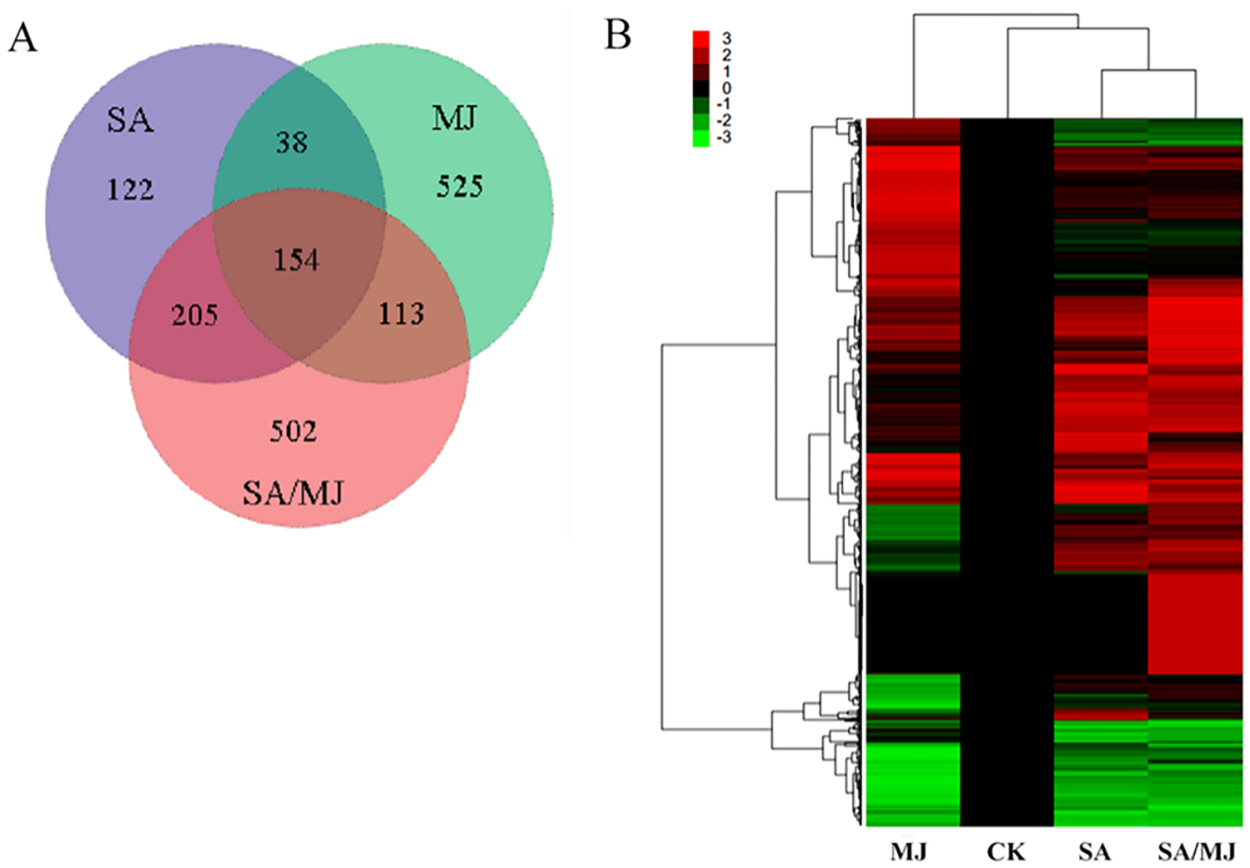
The DEG number and expression profile clustering analysis. **(A)** Venn diagram of the DEGs. The number of DEGs in the SA, MJ and SA/MJ treatment groups were relative to those in the CK group. **(B)** Heat map of the DEGs after pairwise comparisons between the four different groups. Each column represents one treatment group, and each row represents one unigene. The colors indicate the expression values scaled to standard deviations and centered at the normal intensity level (Z-score). Red indicates increased expression relative to the control conditions, and green indicates decreased expression.

The CK-vs-SA, CK-vs-MJ, and CK-vs-SA/MJ pairwise comparisons showed that 519, 830, and 974 differentially expressed unigenes were screened, respectively (Fig 5A). The three hormone treatment groups had 154 unigenes in common. A hierarchical cluster analysis of the expression profiles of 2,022 DEGs from the pairwise comparisons was performed. The gene expression pattern in the SA group was similar to that of the SA/MJ group, whereas it was substantially different from that of the MJ group (Fig 5B).

#### DEG analysis in the SA treatment group

Five hundred and nineteen DEGs were identified in response to SA, including 374 up-regulated and 145 down-regulated unigenes. To generate an overview of the SA-responsive DEGs, we performed a GO analysis. The GO enrichment analysis revealed that 7 significantly enriched GO terms in the DEGs responded to SA. In the “cellular component” domain, the major categories included “non-membrane-bounded organelle”, “intracellular non-membrane-bounded organelle”, and “ribonucleoprotein complex”; in the “molecular function” domain, the significant subcategories were “structural molecule activity” and “structural constituent of ribosome”. No GO terms were identified in the “biological process” domain (S2 Table).

To identify the biological pathways that were active after the SA treatment, we mapped 519 DEGs to canonical signaling pathways in the KEGG database. Twelve pathways were significantly enriched (*P*<0.05). Among them, numerous genes were involved in metabolism-related pathways, including “alpha-Linolenic acid metabolism”, “Cyanoamino acid metabolism”, “Starch and sucrose metabolism”, “Pentose and glucuronate interconversions”, “Galactose metabolism”, “Photosynthesis” and “Photosynthesis-antenna proteins”. Other DEGs were enriched in the “Phagosome”, “Ribosome”, “ABC transporters”, “mRNA surveillance”, and “Betalain biosynthesis” pathways.

#### DEG analysis in the MJ treatment group

Among the 830 differentially expressed unigenes following MJ treatment, 528 genes were up-regulated, and 302 were down-regulated. Five hundred and twenty-five unigenes were unique to the MJ treatment, which exceeded the number in the SA group (122 unigenes). This difference suggested that MJ might induce more genes in response to high temperature than those of SA. The GO enrichment showed that 26 GO terms were significantly enriched in response to MJ. In the “cellular component” category, the 3 most significant subcategories were “ribosome”, “ribonucleoprotein complex”, and “non-membrane-bounded organelle”; in the “molecular function” category, the enrichment subcategories were identical to those in the SA group. In contrast to the SA group, multiple GO terms were significantly overrepresented in the “biological process” category, including “translation”, “cellular macromolecule biosynthetic process”, and “gene expression” in the MJ group (S3 Table).

Based on the KEGG enrichment analysis, the DEGs in the MJ group were highly enriched in 11 pathways (*P*<0.05). Similar to the SA results, numerous genes were involved in metabolism-related pathways, including “Starch and sucrose metabolism”, “Pentose and glucuronate interconversions” “Glyoxylate and dicarboxylate metabolism”, “Nitrogen metabolism”, “Photosynthesis”, “Glutathione metabolism”, “Glycine, serine and threonine metabolism”, “Vitamin B6 metabolism” and “Synthesis and degradation of ketone bodies”. The “Ribosome” and “Phagosome” pathways were also altered by MJ.

#### DEG analysis in the SA/MJ treatment group

There were 974 DEGs between the control and the SA/MJ treatment, of which 821 unigenes were up-regulated, and 153 unigenes were down-regulated. More DEGs, particularly up-regulated genes, were identified in the SA/MJ group than those in the SA or MJ group, which suggested that the combined SA and MJ could induce expression in a greater number of genes to improve heat tolerance in *Gp*. *lemaneiformis*. The GO enrichment showed 41 significantly enriched GO terms for the SA/MJ-responsive DEGs. In the “cellular component” category, the most significant subcategories were “endoplasmic reticulum”, “plasma membrane”, and “cell periphery”. The major enrichment subcategories of “molecular function” were “2-alkenal reductase [NAD(P)] activity”, “peroxidase activity”, and “oxidoreductase activity, acting on peroxide as acceptor”. In “biological process”, 20 stress-related subcategories were significantly enriched, including “response to stress”, “protein folding”, and “response to heat” (S4 Table).

Ten pathways were significantly enriched (*P*<0.05) in the KEGG enrichment analysis. Numerous genes were highly enriched in pathways that related to genetic information processing, including “Ribosome biogenesis in eukaryotes”, “Protein processing in endoplasmic reticulum”, and “Ribosome”. Multiple DEGs were enriched in “Biosynthesis of secondary metabolites” (e.g., “Phenylpropanoid biosynthesis”, “Flavonoid biosynthesis” and “Phenylalanine biosynthesis). Other DEGs were enriched in “Cyanoamino acid metabolism”, “Cutin, suberine and wax biosynthesis”, “alpha-Linolenic acid metabolism”, and “Phagosome”.

#### Analysis of crosstalk between SA and MJ

To further investigate the combined effects of SA and MJ in enhancing thermotolerance in *Gp*. *lemaneiformis*, we analyzed the significance of the expression profile of each unigene in the SA/MJ group, using the SA and MJ groups as controls. A total of 240 genes in the SA/MJ treatment group exhibited significantly different expression patterns from those in the SA and MJ treatment groups. Among these genes, 216 were synergistically and 11 were antagonistically regulated by SA and MJ, respectively. Additionally, 7 genes showed opposing expression changes in response to SA and MJ treatments, which suggested that these genes were also antagonistically regulated by SA and MJ (S5 Table).

**Fig 6:**
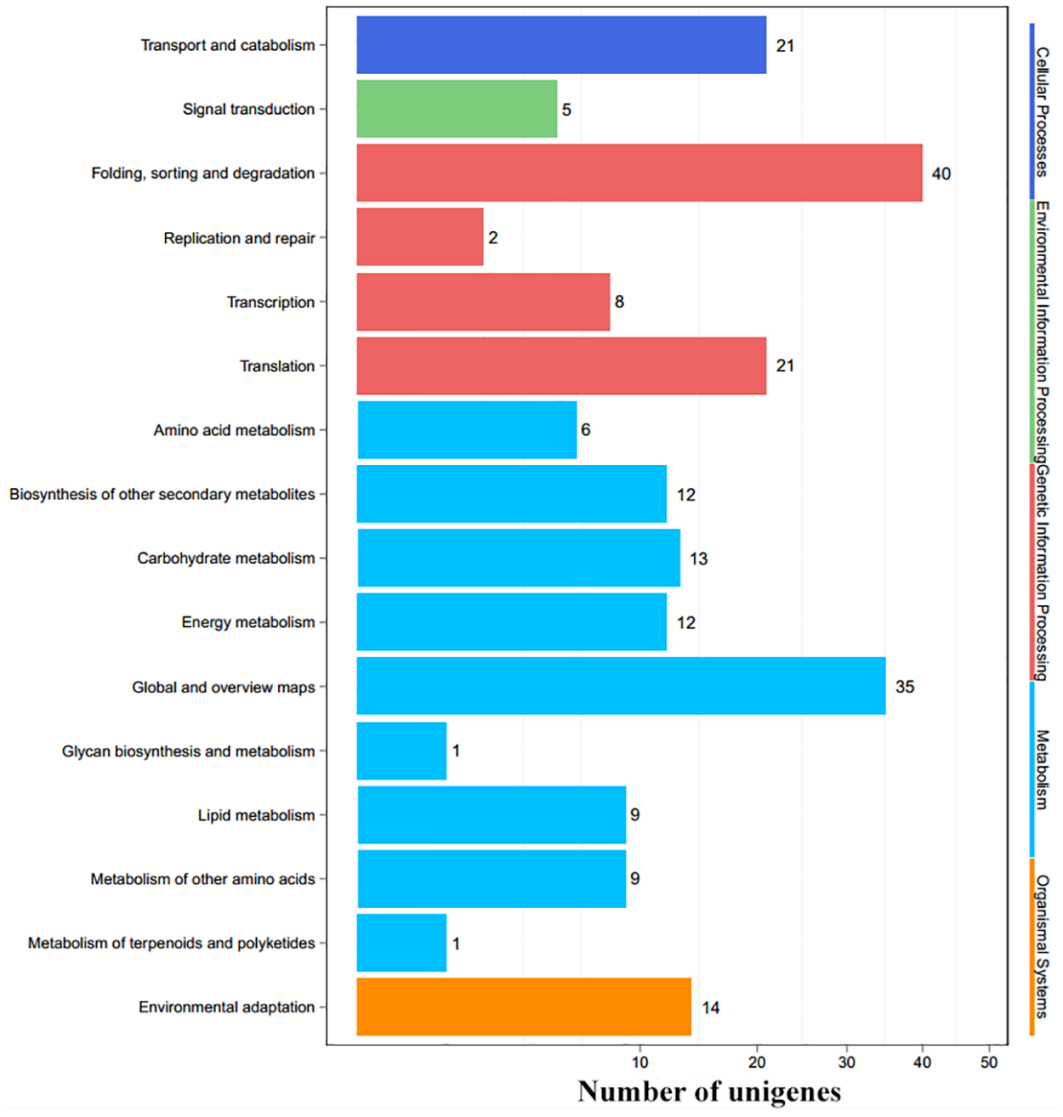
KEGG analysis of the synergistic genes regulated by SA and MJ in *Gp*. *Lemaneiformis*.

To explore the synergistically modulated biological processes and pathways between SA and MJ under heat stress, we analyzed the 216 synergistic genes using GO and KEGG enrichment analyses. Thirty-five GO terms in biological process were significantly enriched (*P*<0.05) (S6 Table). Predictably, some unigenes were involved in stress responses such as “protein folding”, “response to heat”, and “response to hydrogen peroxide”. The top 4 pathways in the KEGG analysis included “Folding, sorting and degradation” (40), “Global and overview maps” (35), “Transport and catabolism” (21), and “Translation” (21). Moreover, other pathways of the synergistic genes were associated with “Carbohydrate metabolism” (13), “Biosynthesis of secondary metabolites” (12), “Energy metabolism” (12), “Environmental adaptation” (5) (Fig 6). Most of the 18 antagonistic genes consisted of unknown proteins.

### Specific DEGs in response to the two phytohormones

#### Photosynthesis-related DEGs

The KEGG enrichment analysis results indicated that exogenous SA and MJ improved photosynthesis during heat stress (S7-I Table). However, the effects of exogenous SA and MJ on photosynthesis were not identical. Regarding the light reaction, photosystem I (PSI)-associated genes, including four PsaO genes and two chlorophyll a/b binding protein (CL1246.Contig2_All, Unigene3077_All) genes, were significantly induced by SA. In contrast to the SA group, the MJ treatment resulted in up-regulation of transcripts that were associated with photosystem II (PSII) (e.g., cytochrome c550, and CP47 chlorophyll apoprotein) and one down-regulated gene that encoded chlorophyll a/b binding protein (CL690.Contig1_All). Other genes that encoded PSI subunit VII, cytochrome c6, 3-oxoacyl-acyl-carrier-protein synthase, allophycocyanin showed high abundances. In the SA/MJ group, another chlorophyll a/b binding protein (CL1440.Contig2_All), ferredoxin, and two PSII oxygen-evolving enhancer proteins, showed substantially increased abundances. Others genes that encoded chlorophyll a/b binding protein (CL1246.Contig1_All, Unigene8520_All), PSI subunit III, cytochrome b6, phycobilisome linker polypeptide and phycoerythrin beta subunit were also induced by combination of SA and MJ. Additionally, transcripts of PSII P680 reaction center D1 protein were induced by SA and MJ, while phycobilisome linker polypeptide and cytochrome b6 showed increased abundances by MJ and SA/MJ.

In the Calvin cycle, pyruvate kinase (PK) was up-regulated, while glyceraldehyde-3-phosphate dehydrogenase (GAPDH) and two fructose-bisphosphate aldolase (FBA) genes were down-regulated by MJ. The SA/MJ treatment dramatically up-regulated two ribulose-bisphosphate carboxylase small chain genes and slightly down-regulated one pyruvate, orthophosphate dikinase gene. In the MJ and SA/MJ groups, two carbon fixation-associated genes were induced, including malate dehydrogenase (MDH) and the ribulose-1, 5-bisphosphate carboxylase/oxygenase large subunit (rbcL). Meanwhile, rbcL was also simultaneously induced by SA.

#### Glycometabolism-related DEGs

The KEGG enrichment analysis results indicated that there were significant changes of the glycometabolism-related transcripts in the SA, MJ and SA/MJ groups (S7-II Table). In the “Glycolysis/Gluconeogenesis” pathway, genes that encoded GAPDH (CL492.Contig3_All), glucokinase (GK), phosphoglucomutase (PGM) and acetyl-CoA synthetase (ACS) were induced by SA. In the MJ group, transcripts for aldose 1-epimerase (AEP), PK, pyruvate dehydrogenase E1 and E2 components, and glucose-6-phosphate 1-epimerase (G6P-1-E) showed increased abundances, but genes that encoded FBA and GAPDH (CL874.Contig1_All) were down-regulated. Transcripts for 4 GAPDH, alcohol dehydrogenase, ACS and pyruvate dehydrogenase E2 components were induced by SA/MJ.

In the “Starch and sucrose metabolism” pathway, one UDP glucose 6-dehydrogenase (UGDH), and an isoamylase gene were both up-regulated, and one maltase-glucoamylase (MGA, Unigene1125_All) gene was down-regulated by all three treatments. However, another MGA (CL780.Contig2_All) was solely induced by MJ. Transcripts for PGM and GK were specifically induced by SA, and a starch branching enzyme was specifically down-regulated by MJ. Two beta-glucosidase genes were highly induced by SA/MJ. We also observed enhanced transcript abundances for 6-phosphogluconate dehydrogenase in the MJ group in the “Pentose phosphate pathway.

Additionally, several glycoside hydrolases (GHs) and glycosyltransferases (GTs) were detected. Three alpha-glucosidase families GH31 were induced by SA, and one CesA-like cellulose synthase from family GT2 and one cellobiohydrolase A were solely induced by MJ. Moreover, almost all the GT23 family members showed increased abundances in the MJ and SA/MJ groups. Only one down-regulated family GH16 endohydrolysis of (1–4)-beta-D-linkages of galactans, was found in the SA and SA/MJ treatments.

#### Protein synthesis-related DEGs

In addition to several unknown proteins, we found that most of the down-regulated genes were ribosomal proteins (S7-III Table). Except for the 60S acidic ribosomal protein P0 and the ribosomal protein S10, which were up-regulated by SA, and 12 DEGs of 40S or 60S ribosomal proteins, which were up-regulated by SA/MJ, the other DEGs were all down-regulated. In these down-regulated ribosomal proteins, 41 were regulated by MJ alone, and the other unigenes were affected by either SA and MJ (10), or by MJ and SA/MJ (9), and the remaining ribosomal proteins (6) were altered by all three treatments. Thus we deduced that the ribosomal proteins were predominantly down-regulated by MJ.

In the contrary, although several translation-related unigenes were repressed, almost all other translation initiation factors and translation elongation factors were induced in the three treatment groups. Relatively speaking, more translation-related unigenes were affected by MJ or SA/MJ than those by SA.

#### Heat shock-related DEGs

Similar to the results of ribosomal proteins, almost all the heat shock proteins (Hsps) or chaperones were detected in the MJ and SA/MJ groups (S7-IV Table). Almost all DEGs in the SA/MJ group were substantially up-regulated. These DEGs induced by SA/MJ consisted of 4 from the Hsp100 family, 8 from the Hsp90 family, 12 from the Hsp70 family, 4 from the DnaJ homolog subfamily, 5 from the Hsp60 family and 16 small heat shock proteins (sHsp). In contrast to the SA/MJ treatment, one Hsp100, 3 Hsp70, 4 DnaJ homologs, and one co-chaperonin GroES (Hsp10) were down-regulated, and only two were up-regulated in the MJ group. These results suggested that the combination of SA and MJ might induce more Hsps genes in response to heat stress than those of SA or MJ.

#### ROS-related DEGs

The GO term enrichment analysis showed that the “peroxidase activity” and “antioxidant activity” subcategories of “molecular function” were significantly activated by SA/MJ. To further characterize the differences in ROS-related gene expression between the treatments and the control, we identified 35 genes that encoded ROS scavengers (S7-V Table). Four vanadium-dependent bromoperoxidase (vBPO) genes were significant induced only in the SA group. Three L-ascorbate peroxidase (APX) genes, a glutathione S-transferase (GST) (Unigene2760_All) and a NADPH oxidase, respiratory burst oxidase homologue (Narboh) were solely induced by MJ, while another GST (CL1021.Contig1_All) displayed the opposite expression pattern. In the SA/MJ treatment, 3 APX, 2 GST, 2 catalase (CAT) and 3 peroxidase transcripts were all markedly up-regulated. Additionally, transcripts that encoded respiratory burst oxidase homolog (Rboh), GST and peroxiredoxin Q/BCP were all up-regulated by the three treatments. In conclusion, more ROS-related unigenes were dramatically induced by SA/MJ compared to those induced by SA or MJ alone.

#### Calcium signaling-associated DEGs

There were significant changes to signal transduction-related transcripts by the two phytohormones. Calcium is an important second messenger in plants during heat stress. In this study, 0, 4 and 9 calcium regulation-associated genes were affected by SA, MJ and SA/MJ alone under heat stress, respectively (S7-VI Table). One calreticulin gene (Unigene7636_All) was down-regulated by all three treatments, but another calreticulin (Unigene8585_All) was strongly induced by SA/MJ. A calcium-dependent protein kinase (CDPK) was up-regulated by both SA and SA/MJ. Two calcineurin B-like protein (CBL)-interacting serine/threonine-protein kinase unigenes (Unigene1314_All, Unigene4206_All) were induced by MJ, and the other two (Unigene8320_All, CL617.Contig3_All) were induced by SA/MJ. One Ca^2+^/calmodulin-dependent protein kinase (Unigene3650_All) was down-regulated by MJ, and another (Unigene6144_All) was induced by SA/MJ. Two calmodulin (CaM) genes showed highly increased abundances in the SA/MJ group. Moreover, genes that encoded calnexin, CaM, calcium ion-binding protein, and calcyclin-binding protein were induced by SA/MJ.

#### Phytohormone-associated DEGs

The analysis revealed that 2, 7, 9 hormone-related genes were activated by SA, MJ or SA/MJ under heat stress, respectively (S7-VII Table). Only one unigene from the abscisic acid signaling pathway that encoded abscisic acid insensitive 4 (ABI4) was induced by all three treatments. In the auxin signaling pathway, transcriptional expression of 4 genes increased, including two auxin efflux carriers (AEC) in the MJ group, an auxin-responsive protein IAA9 and an auxin-repressed protein-like protein ARP1 in the SA/MJ group. In the ethylene signaling pathway, one serine/threonine-protein kinase (CTR1) gene (Unigene5660_All) was down-regulated by SA. In contrast, two CTR1 genes (Unigene544_All, Unigene7283_All) were slightly induced by MJ. Moreover, two genes that encoded ethylene insensitive 3 (EIN3) were significantly induced by SA/MJ. In the cytokinin signal pathway, one down-regulated gene that encoded a two-component response regulator ARR-B family and one up-regulated gene that encoded an ompR transcriptional regulator were specifically identified in the MJ group. Additionally, two up-regulated genes of brassinosteroid insensitive 1 and arabidopsis histidine kinase 2/3/4 (cytokinin receptor), and one down-regulated spermine oxidase gene were specifically detected in the SA/MJ group.

### DEGs validation by qPCR

To confirm the accuracies and reliabilities of the expression profiles revealed by RNA-seq, we monitored the expression of 30 candidate genes in the SA, MJ and SA/MJ groups by qPCR. These candidate DEGs included 6 photosynthesis-related genes, 6 stress-related genes, 8 glycometabolism-associated genes, 4 lipid metabolism-associated genes, 3 hormone-related genes and 3 unrelated genes. Our results indicated that approximately 90.00% of the genes showed similar expression patterns between the RNA-seq and qPCR analyses (S8 Table). Specifically, the expression levels of 26 genes in 59 treatments (65.56%) according to the qPCR assay showed good consistencies with the RNA-seq results (Class I). Their fold changes fell between 0.5- and 2- fold the 2-fold; the expression levels of 16 genes in 22 treatments (24.44%) by qPCR differed from those detected by RNA-seq with greater than 2-fold or lower than 0.5-fold differences (Class II). However, 8 genes in 9 treatments (10.00%) displayed clear opposing expression tendencies that were revealed by the qPCR and RNA-seq analyses (Class III). In summary, the qPCR verification demonstrated that our RNA-seq results were reliable.

The qPCR results of these candidate genes were shown in S2 Fig. In one example, the vBPO transcript was induced 2.44-, 2.17- and 2.22-fold relative to that of the control by SA, MJ and SA/MJ, respectively. The RNA-seq data also demonstrated that vBPO expression increased in the three groups, but with a slightly higher fold change. In the 2^nd^ case, the expression of AEC was induced approximately 1.92-fold with the MJ treatment. However, it was markedly increased 6.97-fold by MJ in the RNA-seq data. In the 3^rd^ case, opposing expression profiles were observed in the qPCR and transcriptome data. For example, the CTR1 increased its expression 4.57- and 6.48-fold relative to that of the control in the MJ and SA/MJ groups, respectively; however, its expression decreased with both treatments in the RNA-seq data.

## Discussion

The effects of heat stress have been documented in *Gp*. *lemaneiformis*. For example, a 2-dimensional gel electrophoresis (2-DE) proteomics analysis revealed the involvement of thirteen proteins in heat tolerance, including proteins that are associated with photosynthesis, energy metabolism, protein-folding catalysis, transcription, and molecular chaperoning [16]. The transcriptomic results of our study revealed various pathways that participated in SA- and MJ-induced heat resistance.

### Significant metabolic pathways regulated by SA and MJ

Numerous genes exhibited significant expression changes after exposure to SA and MJ during heat stress in *Gp*. *lemaneiformis*. Most transcripts of the 192 commonly regulated DEGs by SA and MJ were related to photosynthesis, glycometabolism, ROS scavenging and signal transduction.

The adverse effects of heat stress on photosynthesis have been attributed to impaired structural organization and inhibited electron transport and PSII activity [17,18]. In this study, multiple up-regulated transcripts that correlated with PSI and PSII were detected in the SA and MJ groups. PSI-related genes were stimulated by SA, but more PSII genes were promoted by MJ. The D1 protein plays key roles in maintaining PSII activity, and its encoding gene, psbA, was induced by SA and MJ. The PSII recovery mechanism included the removal of damaged D1 proteins and their replacement with newly synthesized molecules. These results indicated that SA and MJ alleviated heat stress-induced damage to the photosystem and accelerated the restoration of photosynthetic function. SA and MJ also counteracted the reduced CO_2_ fixation rate under stress conditions [19,20]. Together with the Rubisco large subunit, other genes in the Calvin cycle, including PK and MDH, were up-regulated after the MJ treatment.

Stimulatory effects by SA and MJ on glycometabolism were also observed. In the starch and sucrose metabolic pathway, the genes encoding UGDH and isoamylase were up-regulated by all three treatments. UGDH is a key enzyme that converts UDP-glucose into UDP-glucuronic acid, and UDP-glucose is a direct and indirect precursor of starch and sucrose. Isoamylase, a type of direct debranching enzyme, is involved in starch degradation. However, one starch synthesis-associated branching enzyme was down-regulated by MJ. Together, these observations suggest that SA and MJ may activate starch degradation under heat stress.

ROS can be rapidly produced during heat stress, which leads to multiple downstream events, such as membrane lipid peroxidation, oxidation of proteins and nucleic acids in plants [21]. ROS are also important signaling molecules that activate the ROS-scavenging system, which is vital to maintaining cellular homeostasis. NADPH oxidases are the primary enzymes for stress resistance; they trigger the generation of the oxidative burst [22]. We observed that NADPH oxidase, respiratory burst oxidase homolog was uniquely induced by MJ, and that respiratory burst oxidase homologs were were up-regulated by SA, MJ and SA/MJ. SA and/or MJ increase heat tolerance by increasing the expression or activities of antioxidant enzymes, such as SOD, CAT, and APX [5,6]. In this study, the vBPO expression was exclusively enhanced by SA; several APX expression levels were promoted by MJ and SA/MJ; and CAT, GST and peroxidase transcripts abundances were increased in the SA/MJ group. Thus, SA and MJ activate the ROS-scavenging system to maintain the intracellular redox balance and enhance resistance to heat stress.

Phytohormone signal transduction is an important element of the stress system. CTR1, a protein kinase with sequence similarity to the catalytic domain of the RAF protein kinase (a mitogen-activated protein kinase kinase kinase), is located downstream from ETR1 and functions as a negative signaling regulator [23]. Three CTR1-encoding genes displayed different expression patterns by SA and MJ in the ethylene signaling pathway. Overexpression of *OsPIN3t*, an AEC gene, resulted in increased drought tolerance in rice [24], two auxin efflux carrier-encoding genes were induced by MJ in this study. Sucrose nonfermenting-1 (SNF1)-related protein kinases (SnRKs), which belong to the serine/threonine-protein kinase class have been implicated in stress and abscisic acid responses [25]. Abscisic acid insensitive 4 (ABI4) plays a key role in transmitting information on the abundance of ascorbate and in enhancing cellular protection against oxidative challenges [26]. In our study, one SnRK gene was suppressed by MJ, and one ABI4 gene was induced by three treatments. These data indicate that the ethylene, auxin and abscisic acid signaling pathways participate in SA- and MJ-mediated heat resistance.

### The interactions between SA and MJ

Previous studies have demonstrated that SA- and JA-dependent defense signaling pathways are antagonistic or synergistic with each other [27,28]. Salicylic acid 2-*O*-β-D-glucose (SAG) shows synergistic activity in the JA-related defense response in terms of the MDA, PPO and PAL activities in pea seedlings [29]. We observed synergism in pathways related to energy metabolism, protein folding and degradation, ROS-scavenging enzymes, signaling transduction, and secondary metabolism. Here, we predominantly focused on the genes that were likely to be involved in heat stress resistance. Among the ROS scavengers, multiple antioxidant enzymes, including CAT, APX, Cu/Zn-SOD, POX, and GST, showed enhanced expression. Hsps were previously shown to be involved in conferring basal thermotolerance to plants during heat stress [30,31]. The Hsp family genes constitute the largest SA/MJ-induced family during heat stress. The expression levels of almost all members of the Hsp100, Hsp90, Hsp70, Hsp70 co-chaperone, Hsp60 and small Hsp families were substantially increased. The different Hsp/chaperone classes likely cooperate in cellular protection and play complementary and overlapping roles toward protecting proteins from heat stress.

In plants, intracellular Ca^2+^ levels are modulated in response to heat stress [32]. Exogenous SA increases cytoplasmic Ca^2+^ and Ca^2+^-ATPase activity to maintain Ca^2+^ homeostasis during heat stress and enhances heat tolerance in plants [6]. Specific calcium signatures can be sensed and transmitted to downstream responders by different Ca^2+^ sensors, including CaMs, CBLs, and CDPKs [33,34]. Data on CaM and CBL expression suggest that they are involved in various stress-induced signaling pathways. In grapes, the exogenous SA-induced thermotolerance is associated with the Ca^2+^-CaM system [35]. CBL5 may positively regulate of salinity or osmotic stress responses in plants [36]. In this analysis, multiple candidate genes encoding the components of calcium- or calmodulin-mediated signaling pathways, including calnexin/calreticulin, CaM, CBL-interacting serine/threonine-protein kinase, and calcium ion-binding protein, were synergistically regulated by SA and MJ, which suggested that Ca^2+^ modulated resistance to heat stress through SA and MJ-regulated signal transduction.

Hormone-related genes were also identified and affected by SA and MJ. Auxin-repressed protein-like protein ARP1, an activator of disease resistance, was synergistically responsive to SA and MJ. ARP1/GERI1 recruits the NPR1 gene, which is essential for SA-mediated defenses, to co-regulate disease resistance [37]. EIN3 is a primary transcription factor downstream of EIN2 that is necessary and sufficient to induce most ethylene response genes and is believed to be involved in ubiquitin-mediated proteolysis [38]. Other genes, including brassinosteroid insensitive 1 and arabidopsis histidine kinase 2/3/4 (cytokinin receptor), which are involved in the brassinosteroid and cytokinin signaling transduction pathways, respectively, were also synergistically regulated by SA and MJ.

Antagonism between JA and SA has also been reported. SA strongly antagonizes the JA signaling pathway by down-regulating JA-responsive genes, such as PDF1.2, AOS, LOX2, and OPR3 [39]. JA functions as an inducer of basic PR genes and as an inhibitor of acidic PR genes, while SA does the opposite [40]. In this study, we identified 18 antagonistic DEGs between SA and MJ and divided them into two classes. One class included 11 genes, for which the SA and MJ combination generated a lower induction relative to that by SA or MJ alone. In this class, we identified two animal heme peroxidase homolog and one respiratory burst oxidase homolog gene. Another class contained 7 genes that were down-regulated by SA but up-regulated by MJ. Only one RNA-directed DNA polymerase homolog was identified in this class. And all the other antagonistic genes were unknown proteins. Interestingly, genes that were up-regulated by SA and also down-regulated by MJ were not observed.

## Conclusions

This study demonstrated that the RNA-seq-based combination of *de novo* transcriptome assembly and digital gene expression profile analysis was powerful for identifying candidate genes and key metabolic processes. More than 2/3 (71.71%) of the unigenes were annotated from 14,644 assembly sequences; 1,659 were differentially expressed in response to SA and/or MJ. The two phytohormones had various effects on gene expression in *Gp*. *lemaneiformis* under heat stress. Relative to SA, MJ and SA/MJ affected more unigenes, such as a large number of ribosomal proteins, translation-related proteins, hsp family, and ROS- and calcium signaling-associated unigenes. Although most the ribosomal proteins were down-regulated, the majority of the other DEGs were up-regulated, especially by the combination of SA and MJ. Thus, SA and MJ co-treatment provoked a stronger response to heat stress relative to that of SA or MJ alone, and synergistic effects were the major mode of action mode between SA and MJ under heat stress. This study may serve as a useful resource for understanding SA- and/or MJ-induced heat tolerance mechanisms in algae.

## Supporting information

S1 Fig Size distribution of all the assembled unigenes. (TIF)

S2 Fig Expression analysis of 30 candidate genes by qPCR. The relative gene expression levels were calculated as the value of SA/CK, MJ/CK, and (SA/MJ)/CK. Errors bars represent the standard deviations for two independent experiments, each with three technical replicates. Letters (a, b, c, d) above the bars indicate significant differences between the respective values (*P* < 0.05).
(TIF)

S1 Table The primers used for DEG validation by qPCR. (DOC)

S2 Table Significantly enriched GO terms of the DEGs in response to SA under heat stress. (DOC)

S3 Table Significantly enriched GO terms of the DEGs in response to MJ under heat stress. (DOC)

S4 Table Significantly enriched GO terms of the DEGs in response to SA/MJ under heat stress. (DOC)

S5 Table The DEGs synergistically and antagonistically regulated by SA and MJ. (XLS)

S6 Table Significantly enriched GO terms in biological process of the synergistic DEGs. (DOC)

S7 Table DEGs involved in different pathways in the phytohormone treatments. (DOC)

S8 Table Comparison of the DEG expression pattern between RNA-seq and qPCR analysis. (DOC)
