# Supplementary figures and images for: Comparative transcriptional profiling of Gracilariopsis lemaneiformis in response to salicylic acid- and methyl jasmonate-mediated heat resistance
Source: PLoS One. 2017 May 2;12(5):e0176531. doi: 10.1371/journal.pone.0176531 (PMC5413009; doi:10.1371/journal.pone.0176531)

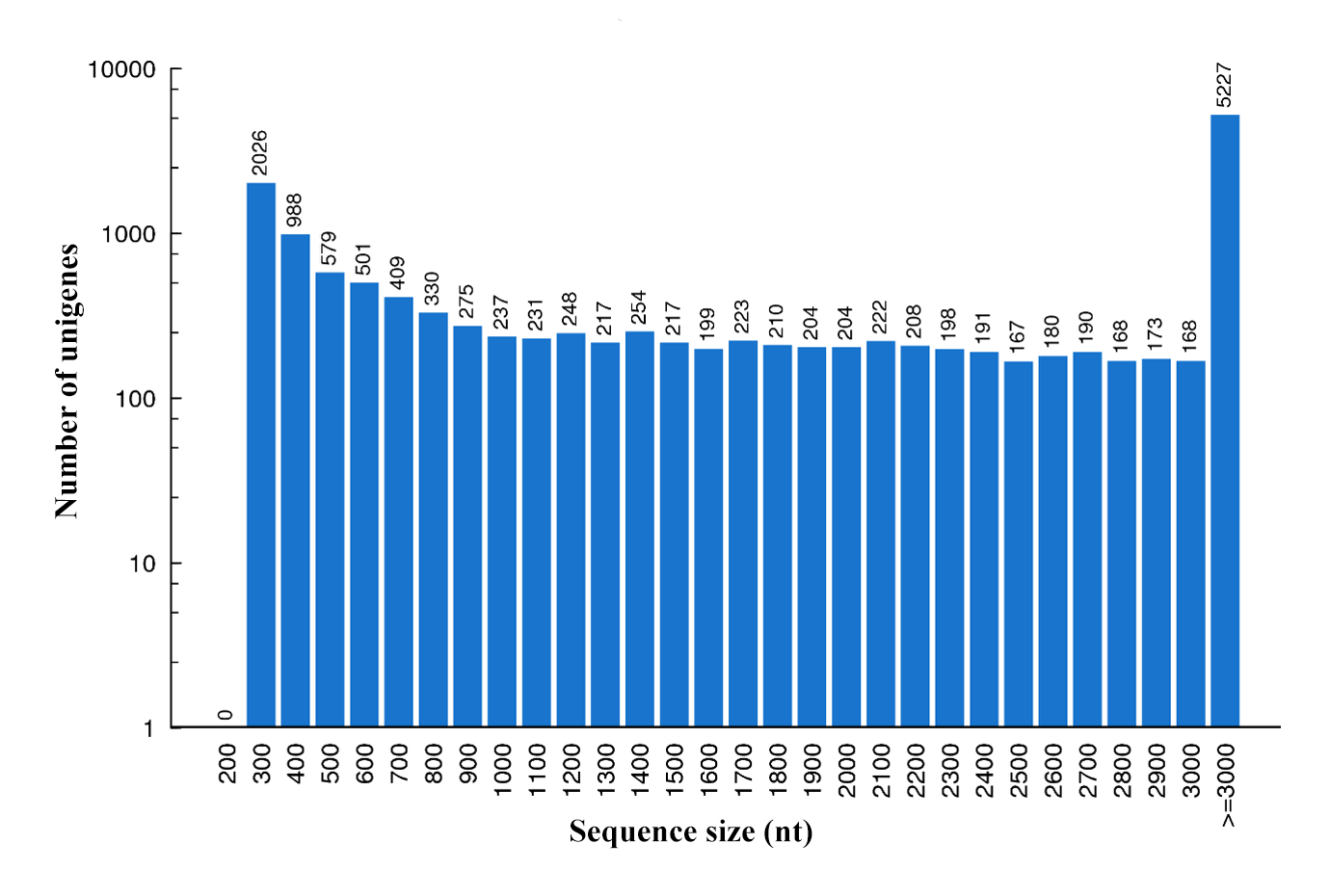

Supplement: S1 Fig — (TIF) [file pone.0176531.s001.tif]

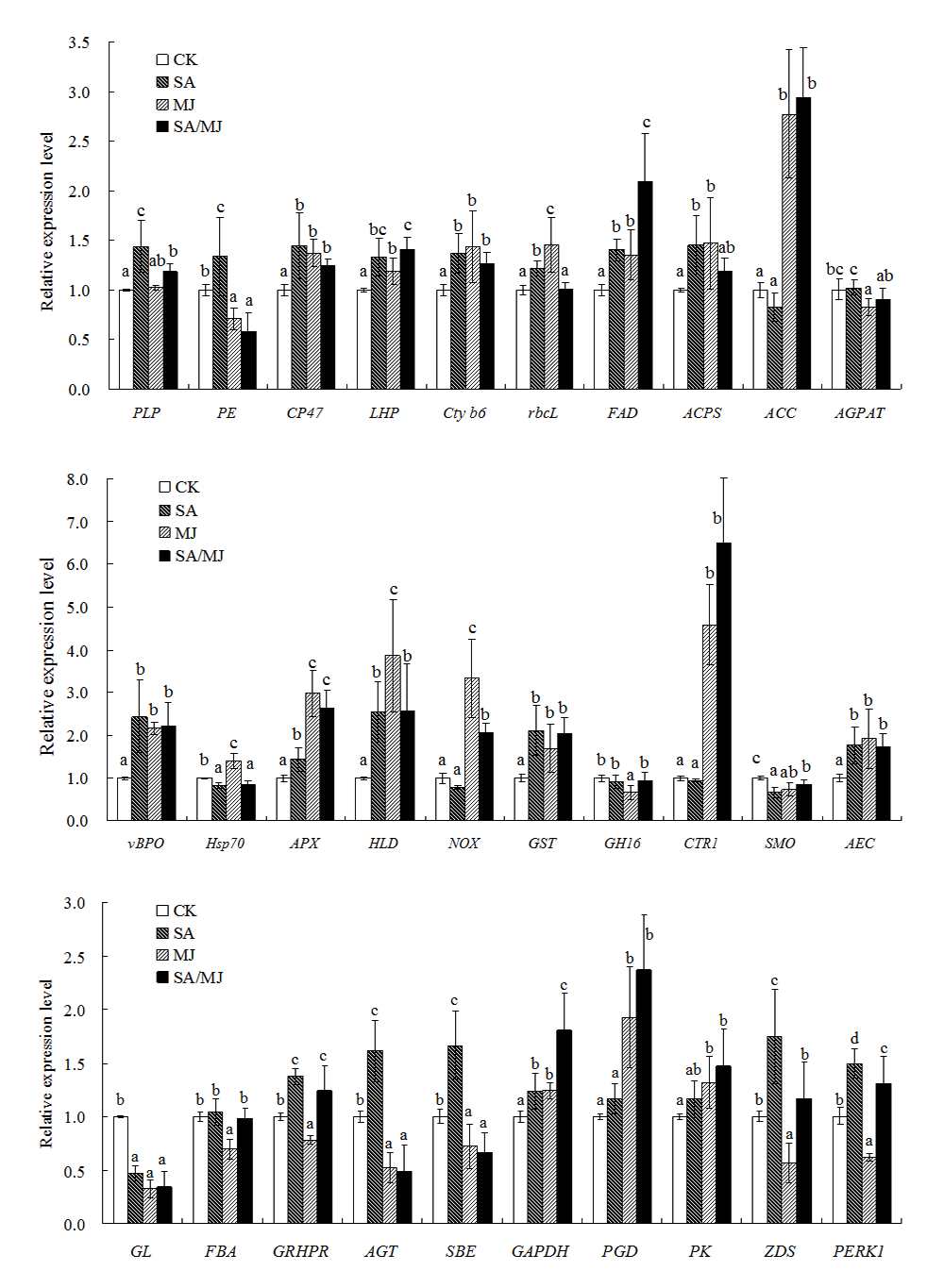

Supplement: S2 Fig — The relative gene expression levels were calculated as the value of SA/CK, MJ/CK, and (SA/MJ)/CK. Errors bars represent the standard deviations for two independent experiments, each with three technical replicates. Letters (a, b, c, d) above the bars indicate significant differences between the respective values (P < 0.05). (TIF) [file pone.0176531.s002.tif]
