# Supplementary material for: Comparative transcriptional profiling of Gracilariopsis lemaneiformis in response to salicylic acid- and methyl jasmonate-mediated heat resistance
Source: PLoS One. 2017 May 2;12(5):e0176531. doi: 10.1371/journal.pone.0176531 (PMC5413009; doi:10.1371/journal.pone.0176531)
Supplement: S1 Table — (DOC) [file pone.0176531.s003.doc]

S1 Table. The primers used for DEG validation by qPCR

| Gene ID | Gene annotation | Gene abbreviation | Primer sequences (5'-3') |
| --- | --- | --- | --- |
| Unigene93_All | phycobilisome linker polypeptide | PLP | GCGGCTTAGATGGTTTGAT  ATTTCGAGCTTCCTGTGGT |
| Unigene5649_All | phycoerythrin beta subunit | PE | AGGAATGATTTGTGAAAACCCAG  AACGGTCTTCTAAAACAGATGGA |
| Unigene6851_All | photosystem II CP47 chlorophyll apoprotein | CP47 | GAGACCCAAGAACAGGAGAAC  TATGAAATGCACCGAAACCAA |
| Unigene3077_All | light-harvesting complex I chlorophyll a/b binding protein 1 | LHP | GCTGCTACGCTCTGAAACAAAC  ACATCTGATGAATCATGCCACC |
| Unigene5514_All | cytochrome b6 | Cytb6 | AAGAGGTGGTGTAAGTGTAGGGC  CTGAAATACCTTGCTTACGAATC |
| Unigene56_All | ribulose-1,5-bisphosphate carboxylase/oxygenase large subunit | rbcL | AGTAACTCCTGTTGCTTCTGGTGGT  CTTCATTACGAGCCATAACCATAGA |
| CL402.Contig1_All | vanadium-dependent bromoperoxidase 1 | vBPO | AGGGCGAGTTGAACAAGGTG  GCGGGACTGTCATGGAGAAG |
| CL335.Contig1_All | heat shock protein 70 | Hsp70 | TTTTGATTTGGGAGGTGGAAC  AGACGGCGAACGGAACG |
| Unigene2196_All | ascorbate peroxidase | APX | CTTCCTTCGCCAGCACCT  AACTGTATCTGGGTCATCAACG |
| CL1163.Contig1_All | haloalkane dehalogenase | HLD | AACGGCGTGGTCGGCTAAT  GGGATTTCGGCGGTTGTTG |
| Unigene455_All | NADPH oxidase | NOX | CGTGGTGCCGGATACAC  GCCGAAACCGAAGAGCC |
| Unigene2550_All | glutathione S-transferase | GST | TACAGCTACAGAGGCAGCGACAC  ACCAAATCGGCAACAGTGAGC |
| Unigene1125_All | alpha-1,4-glucan lyase isozyme | GL | CAACTTTAAGGGTGCGACTTT  CCAACTGGGATGTAATCATAGAC |
| CL1175.Contig1_All | fructose-bisphosphate aldolase, class I | FBA | ACGCCCTTCCAGTGTCC  CGATGCCGTAGAAATCAGC |
| Unigene7277_All | glyoxylate reductase/hydroxypyruvate reductase | GRHPR | TCTGCCGTCTTCGTCAACC  GGTGCGAGTGCCCATAGAA |
| Unigene2767_All | alanine-glyoxylate aminotransferase | AGT | CCTGGCAGACCAACAACGAG  CTGAGGCGAAGTCCGAAGTA |
| Unigene4281_All | starch-branching enzyme | SBE | GTGAAGGAAACGGCAACTC  TTCTCAACGACGACCAGCT |
| Unigene2463_All | glycoside hydrolase family GH16 endohydrolysis of (1-4)-beta-D-linkages of galactans | GH16 | AAAGACGCCTGATGTTTACGA  CAAGCCAACTTTGATCTTCTCC |
| CL492.Contig3_All | glyceraldehyde -3-phosphate dehydrogenase | GAPDH | CGCCAACATTATTCCGTCTA  GCACCGTCAAGTCAACAACC |
| CL240.Contig1_All | 6-phosphogluconate dehydrogenase | PGD | GGACCCTGCGTCACCTACA  GAATACATCAGCGATTTGAGAAAC |
| CL1486.Contig2_All | pyruvate kinase | PK | ACCGTCGGAAGGGTTGTC  GCTTGCCCGTTCGGATTT |
| CL1283.Contig1_All | spermine oxidase | SMO | TACAATAAGTCCGTAACCTC  TCCACAACTCCTTGACCC |
| CL1424.Contig1_All | auxin efflux carrier | AEC | CAAATATGGGTGCCTGTCG  TAGCAATGCGGCGTTCTCA |
| Unigene5660_All | serine/threonine -protein kinase CTR1-like | CTR1 | AAATGGATGCGTCTAACAGT  GGCGACCAGGTAGTTGTT |
| Unigene567_All | omega-3 fatty acid desaturase | FAD | TGGAACTATGTACGAGGCA  GAAGGTGGTAATGAGGGAT |
| Unigene121_All | 3-oxoacyl-acyl-carrier-protein synthase | ACPS | GTTCTGCTGTTCCCGATTTATG  TCTCCTTTCCTTGATCCCTGTA |
| Unigene5267_All | acetyl-CoA carboxylase carboxytransferase | ACC | GAGAAACTAGGACAGGGAGA  CACAAAATAGCAGCACAAGC |
| Unigene2126_All | 1-acyl-sn-glycerol-3-phosphate acyltransferase | AGPAT | GCCGTCGCCATACTCAT  GCCGTCCTGGATGTTGT |
| Unigene5895_All | zeta-carotene desaturase | ZDS | CGTTATGGCTAAAGTGGGC  GCAGCGTTGCGTATCTTG |
| Unigene4373_All | proline-rich receptor-like protein kinase PERK1 | PERK1 | AGATTCATCGCAAAGTCGG  ATTCTGGCGGTGTCATCC |
