# Supplementary material for: Comparative transcriptional profiling of Gracilariopsis lemaneiformis in response to salicylic acid- and methyl jasmonate-mediated heat resistance
Source: PLoS One. 2017 May 2;12(5):e0176531. doi: 10.1371/journal.pone.0176531 (PMC5413009; doi:10.1371/journal.pone.0176531)
Supplement: S2 Table — (DOC) [file pone.0176531.s004.doc]

S2 Table. Significantly enriched GO terms of the DEGs in response to SA under heat stress.

| GO accession | GO term | Corrected *P*-value | DEG number | Background number |
| --- | --- | --- | --- | --- |
| **Cellular component** | | | | |
| GO:0043228 | non-membrane-bounded organelle | 0.00023 | 21 | 495 |
| GO:0043232 | intracellular non-membrane-bounded organelle | 0.00023 | 21 | 495 |
| GO:0030529 | ribonucleoprotein complex | 0.00835 | 16 | 382 |
| GO:0005840 | ribosome | 0.01378 | 15 | 355 |
| GO:0032991 | macromolecular complex | 0.02472 | 20 | 609 |
| **Molecular function** | | | | |
| GO:0005198 | structural molecule activity | 0.00029 | 13 | 276 |
| GO:0003735 | structural constituent of ribosome | 0.00468 | 11 | 257 |

‘DEG number’ means the number of DEGs in this GO function.

‘Background number’ means the number of all unigenes in this GO function.
