# Supplementary material for: Comparative transcriptional profiling of Gracilariopsis lemaneiformis in response to salicylic acid- and methyl jasmonate-mediated heat resistance
Source: PLoS One. 2017 May 2;12(5):e0176531. doi: 10.1371/journal.pone.0176531 (PMC5413009; doi:10.1371/journal.pone.0176531)
Supplement: S3 Table — (DOC) [file pone.0176531.s005.doc]

S3 Table. Significantly enriched GO terms of the DEGs in response to MJ under heat stress.

| GO accession | GO term | Corrected *P*-value | DEG number | Background number |
| --- | --- | --- | --- | --- |
| **Cellular component** | | | | |
| GO:0005840 | ribosome | 9.76e-11 | 65 | 355 |
| GO:0030529 | ribonucleoprotein complex | 3.51e-09 | 65 | 382 |
| GO:0043228 | non-membrane-bounded organelle | 1.69e-07 | 73 | 495 |
| GO:0043232 | intracellular non-membrane-bounded organelle | 1.69e-07 | 73 | 495 |
| GO:0032991 | macromolecular complex | 1.00e-06 | 82 | 609 |
| GO:0022626 | cytosolic ribosome | 0.00122 | 35 | 217 |
| GO:0044391 | ribosomal subunit | 0.00689 | 28 | 170 |
| GO:0044445 | cytosolic part | 0.01875 | 35 | 246 |
| **Molecular function** | | | | |
| GO:0005198 | structural molecule activity | 3.25e-19 | 58 | 276 |
| GO:0003735 | structural constituent of ribosome | 7.67e-17 | 53 | 257 |
| **Biological process** | | | | |
| GO:0006412 | translation | 1.14e-16 | 64 | 357 |
| GO:0034645 | cellular macromolecule biosynthetic process | 1.21e-11 | 66 | 467 |
| GO:0010467 | gene expression | 2.03e-11 | 67 | 484 |
| GO:0009059 | macromolecule biosynthetic process | 7.78e-11 | 66 | 484 |
| GO:0044267 | cellular protein metabolic process | 9.15e-10 | 78 | 670 |
| GO:0019538 | protein metabolic process | 6.77e-09 | 80 | 723 |
| GO:1901576 | organic substance biosynthetic process | 0.00015 | 81 | 891 |
| GO:0044249 | cellular biosynthetic process | 0.00045 | 80 | 895 |
| GO:0009058 | biosynthetic process | 0.00059 | 82 | 932 |
| GO:0008152 | metabolic process | 0.00129 | 143 | 2116 |
| GO:0022900 | electron transport chain | 0.00152 | 11 | 37 |
| GO:0044260 | cellular macromolecule metabolic process | 0.00223 | 82 | 959 |
| GO:0044238 | primary metabolic process | 0.00547 | 114 | 1538 |
| GO:0044237 | cellular metabolic process | 0.00719 | 121 | 1679 |
| GO:0043170 | macromolecule metabolic process | 0.02568 | 85 | 1064 |
| GO:0071704 | organic substance metabolic process | 0.04155 | 118 | 1665 |

‘DEG number’ means the number of DEGs in this GO function.

‘Background number’ means the number of all unigenes in this GO function.
