# Supplementary material for: Comparative transcriptional profiling of Gracilariopsis lemaneiformis in response to salicylic acid- and methyl jasmonate-mediated heat resistance
Source: PLoS One. 2017 May 2;12(5):e0176531. doi: 10.1371/journal.pone.0176531 (PMC5413009; doi:10.1371/journal.pone.0176531)
Supplement: S4 Table — (DOC) [file pone.0176531.s006.doc]

S4 Table. Significantly enriched GO terms of the DEGs in response to SA/MJ under heat stress

| GO accession | GO term | Corrected *P*-value | DEG number | Background number |
| --- | --- | --- | --- | --- |
| **Cellular component** | | | | |
| GO:0005783 | endoplasmic reticulum | 0.00047 | 18 | 57 |
| GO:0005886 | plasma membrane | 0.00117 | 67 | 419 |
| GO:0071944 | cell periphery | 0.01226 | 71 | 483 |
| GO:0005618 | cell wall | 0.01731 | 32 | 169 |
| GO:0030312 | external encapsulating structure | 0.01731 | 32 | 169 |
| GO:0000786 | nucleosome | 0.03124 | 6 | 11 |
| **Molecular function** | | | | |
| GO:0032440 | 2-alkenal reductase [NAD(P)] activity | 5.84e-07 | 15 | 30 |
| GO:0004601 | peroxidase activity | 1.06e-05 | 11 | 19 |
| GO:0016684 | oxidoreductase activity, acting on peroxide as acceptor | 1.06e-05 | 11 | 19 |
| GO:0016209 | antioxidant activity | 1.20e-05 | 13 | 27 |
| GO:0016628 | oxidoreductase activity, acting on the CH-CH group of donors, NAD or NADP as acceptor | 3.13e-05 | 16 | 43 |
| GO:0016491 | oxidoreductase activity | 4.78e-05 | 63 | 397 |
| GO:0016627 | oxidoreductase activity, acting on the CH-CH group of donors | 0.00017 | 16 | 48 |
| GO:0020037 | heme binding | 0.00031 | 14 | 39 |
| GO:0016846 | carbon-sulfur lyase activity | 0.00074 | 5 | 5 |
| GO:0097159 | organic cyclic compound binding | 0.00090 | 136 | 1180 |
| GO:1901363 | heterocyclic compound binding | 0.00090 | 136 | 1180 |
| GO:0046906 | tetrapyrrole binding | 0.00117 | 14 | 43 |
| GO:0005488 | binding | 0.01499 | 173 | 1667 |
| GO:0051082 | unfolded protein binding | 0.03698 | 12 | 44 |
| GO:0016210 | naringenin-chalcone synthase activity | 0.04053 | 4 | 5 |
| **Biological process** | | | | |
| GO:0006950 | response to stress | 2.06e-08 | 77 | 482 |
| GO:0006457 | protein folding | 1.80e-06 | 26 | 92 |
| GO:0010208 | pollen wall assembly | 3.42e-06 | 10 | 14 |
| GO:0010584 | pollen exine formation | 3.42e-06 | 10 | 14 |
| GO:0010927 | cellular component assembly involved in morphogenesis | 3.42e-06 | 10 | 14 |
| GO:0009408 | response to heat | 5.85e-06 | 20 | 60 |
| GO:0009266 | response to temperature stimulus | 0.00035 | 30 | 147 |
| GO:0055114 | oxidation-reduction process | 0.00053 | 55 | 371 |
| GO:0048229 | gametophyte development | 0.00478 | 19 | 80 |
| GO:0009415 | Response to water stimulus | 0.00483 | 11 | 30 |
| GO:0048646 | anatomical structure formation involved in morphogenesis | 0.00507 | 10 | 25 |
| GO:0045229 | external encapsulating structure organization | 0.00584 | 12 | 36 |
| GO:0080110 | sporopollenin biosynthetic process | 0.02039 | 6 | 10 |
| GO:0050896 | response to stimulus | 0.02116 | 92 | 819 |
| GO:0009414 | response to water deprivation | 0.02336 | 10 | 29 |
| GO:0042542 | response to hydrogen peroxide | 0.02665 | 9 | 24 |
| GO:0006333 | chromatin assembly or disassembly | 0.03660 | 7 | 15 |
| GO:0009644 | response to high light intensity | 0.03660 | 7 | 15 |
| GO:1901700 | response to oxygen-containing compound | 0.03946 | 28 | 166 |
| GO:0006952 | defense response | 0.04753 | 20 | 101 |

‘DEG number’ means the number of DEGs in this GO function.

‘Background number’ means the number of all unigenes in this GO function.
