# Supplementary material for: Comparative transcriptional profiling of Gracilariopsis lemaneiformis in response to salicylic acid- and methyl jasmonate-mediated heat resistance
Source: PLoS One. 2017 May 2;12(5):e0176531. doi: 10.1371/journal.pone.0176531 (PMC5413009; doi:10.1371/journal.pone.0176531)
Supplement: S6 Table — (DOC) [file pone.0176531.s008.doc]

S6 Table. Significantly enriched GO terms in biological process of the synergistic DEGs

| GO accession | GO term | Corrected *P*-value | DEG number | Background number |
| --- | --- | --- | --- | --- |
| GO:0006457 | protein folding | 3.44e-12 | 29 | 132 |
| GO:0009408 | response to heat | 1.14e-11 | 27 | 118 |
| GO:0006950 | response to stress | 1.34e-11 | 80 | 937 |
| GO:0009266 | response to temperature stimulus | 5.71e-10 | 39 | 284 |
| GO:0042542 | response to hydrogen peroxide | 1.02e-09 | 19 | 65 |
| GO:0006979 | response to oxidative stress | 1.03e-08 | 27 | 154 |
| GO:1901700 | response to oxygen-containing compound | 2.95e-08 | 41 | 351 |
| GO:0000302 | response to reactive oxygen species | 4.59e-08 | 19 | 79 |
| GO:0009644 | response to high light intensity | 1.72e-07 | 16 | 58 |
| GO:0009628 | response to abiotic stimulus | 1.78e-07 | 62 | 739 |
| GO:0050896 | response to stimulus | 1.97e-07 | 100 | 1571 |
| GO:0009642 | response to light intensity | 5.27e-07 | 17 | 71 |
| GO:0010035 | response to inorganic substance | 1.26e-06 | 49 | 531 |
| GO:0034976 | response to endoplasmic reticulum stress | 2.55e-06 | 14 | 51 |
| GO:0042221 | response to chemical stimulus | 5.55e-06 | 64 | 842 |
| GO:0010208 | pollen wall assembly | 0.00026 | 9 | 27 |
| GO:0010584 | pollen exine formation | 0.00026 | 9 | 27 |
| GO:0010927 | cellular component assembly involved in morphogenesis | 0.00026 | 9 | 27 |
| GO:0006869 | lipid transport | 0.00150 | 6 | 12 |
| GO:0009607 | response to biotic stimulus | 0.00205 | 25 | 236 |
| GO:0051704 | multi-organism process | 0.00522 | 27 | 281 |
| GO:0051707 | response to other organism | 0.00627 | 24 | 235 |
| GO:0009415 | response to water stimulus | 0.00661 | 11 | 58 |
| GO:0006333 | chromatin assembly or disassembly | 0.00775 | 7 | 22 |
| GO:0010876 | lipid localization | 0.01142 | 6 | 16 |
| GO:0009414 | response to water deprivation | 0.03417 | 10 | 57 |
| GO:0055114 | oxidation-reduction process | 0.03502 | 35 | 458 |
| GO:0006323 | DNA packaging | 0.03508 | 6 | 19 |
| GO:0006334 | nucleosome assembly | 0.03508 | 6 | 19 |
| GO:0031497 | chromatin assembly | 0.03508 | 6 | 19 |
| GO:0034728 | nucleosome organization | 0.03508 | 6 | 19 |
| GO:0065004 | protein-DNA complex assembly | 0.03508 | 6 | 19 |
| GO:0071824 | protein-DNA complex subunit organization | 0.03508 | 6 | 19 |
| GO:0009617 | response to bacterium | 0.04271 | 16 | 137 |
| GO:0010286 | heat acclimation | 0.04412 | 7 | 28 |

‘DEG number’ means the number of DEGs in this GO function.

‘Background number’ means the number of all unigenes in this GO function.
