# Supplementary material for: Comparative transcriptional profiling of Gracilariopsis lemaneiformis in response to salicylic acid- and methyl jasmonate-mediated heat resistance
Source: PLoS One. 2017 May 2;12(5):e0176531. doi: 10.1371/journal.pone.0176531 (PMC5413009; doi:10.1371/journal.pone.0176531)
Supplement: S7 Table — (DOC) [file pone.0176531.s009.doc]

S7 Table. DEGs involved in different pathways in the phytohormone treatments.

| Unigene ID | log_2_(SA/CK) | log_2_(MJ/CK) | log_2_[(SA/MJ)/CK] | Gene annotation |
| --- | --- | --- | --- | --- |
| **(I) Photosynthesis-related DEGs** | | | | |
| **light reaction** | | | | |
| CL186.Contig1_All | 1.76 | ns | ns | photosystem I subunit PsaO |
| CL186.Contig2_All | 1.56 | ns | ns | photosystem I subunit PsaO |
| CL186.Contig6_All | 1.59 | ns | ns | photosystem I subunit PsaO |
| CL186.Contig7_All | 1.65 | ns | ns | photosystem I subunit PsaO |
| CL1246.Contig2_All | 1.03 | ns | ns | light-harvesting complex I chlorophyll a/b binding protein 4 |
| Unigene3077_All | 1.06 | ns | ns | light-harvesting complex I chlorophyll a/b binding protein 1 |
| CL690.Contig1_All | ns | -1.19 | ns | light-harvesting complex I chlorophyll a/b binding protein 1 |
| Unigene6732_All | ns | 2.61 | ns | photosystem II cytochrome c550 |
| Unigene6851_All | ns | 2.52 | ns | photosystem II CP47 chlorophyll apoprotein |
| Unigene7168_All | ns | 2.38 | ns | photosystem I subunit VII |
| Unigene4927_All | ns | 1.89 | ns | cytochrome c6 |
| Unigene121_All | ns | 1.82 | ns | 3-oxoacyl-acyl-carrier-protein synthase 3 |
| Unigene4245_All | ns | 1.51 | ns | allophycocyanin alpha subunit |
| CL1440.Contig2_All | ns | ns | 11.59 | light-harvesting complex II chlorophyll a/b binding protein 1 |
| Unigene8520_All | ns | ns | 4.33 | light-harvesting complex II chlorophyll a/b binding protein 5 |
| CL1246.Contig1_All | ns | ns | 1.03 | light-harvesting complex I chlorophyll a/b binding protein 4 |
| Unigene8649_All | ns | ns | 11.16 | ferredoxin, chloroplastic |
| Unigene8529_All | ns | ns | 11.07 | photosystem II oxygen-evolving enhancer protein 1 |
| Unigene8794_All | ns | ns | 10.70 | photosystem II oxygen-evolving enhancer protein 2 |
| Unigene1960_All | ns | ns | 1.31 | photosystem II PsbH protein |
| Unigene6488_All | ns | ns | 1.04 | photosystem I subunit III |
| Unigene5649_All | 1.08 | ns | 1.29 | phycoerythrin beta subunit |
| Unigene7014_All | 1.06 | 3.12 | ns | photosystem II P680 reaction center D1 protein |
| Unigene93_All | ns | 2.80 | 1.48 | phycobilisome linker polypeptide |
| Unigene5514_All | ns | 2.15 | 1.32 | cytochrome b6 |
| **Calvin cycle** | | | | |
| CL1486.Contig2_All | ns | 1.14 | ns | pyruvate kinase |
| CL1175.Contig1_All | ns | -2.63 | ns | fructose-bisphosphate aldolase, class I |
| CL1175.Contig2_All | ns | -3.80 | ns | fructose-bisphosphate aldolase, class I |
| CL874.Contig1_All | ns | -1.42 | ns | glyceraldehyde-3-phosphate dehydrogenase, chloroplast |
| Unigene467_All | ns | ns | -1.05 | pyruvate, orthophosphate dikinase |
| Unigene8561_All | ns | ns | 12.16 | ribulose-bisphosphate carboxylase small chain |
| Unigene8306_All | ns | ns | 11.81 | ribulose-bisphosphate carboxylase small chain |
| CL317.Contig6_All | ns | 1.03 | 1.08 | malate dehydrogenase |
| Unigene56_All | 1.74 | 3.79 | 1.62 | ribulose-1,5-bisphosphate carboxylase/oxygenase large subunit |
| **(II) Glycometabolism-related DEGs** | | | | |
| **Glycolysis/Gluconeogenesis** | | | | |
| CL492.Contig3_All | 1.11 | ns | ns | glyceraldehyde 3-phosphate dehydrogenase |
| Unigene6875_All | 1.63 | ns | ns | glucokinase |
| Unigene97_All | 1.01 | ns | ns | phosphoglucomutase |
| CL322.Contig3_All | 2.68 | ns | ns | acetyl-CoA synthetase |
| CL1084.Contig2_All | ns | 1.29 | ns | aldose 1-epimerase |
| CL1486.Contig2_All | ns | 1.14 | ns | pyruvate kinase |
| Unigene8145_All | ns | 11.42 | ns | pyruvate dehydrogenase E1 component |
| Unigene6084_All | ns | 1.01 | ns | glucose-6-phosphate 1-epimerase |
| CL1175.Contig1_All | ns | -2.63 | ns | fructose-bisphosphate aldolase, class I |
| CL1175.Contig2_All | ns | -3.80 | ns | fructose-bisphosphate aldolase, class I |
| CL874.Contig1_All | ns | -1.42 | ns | glyceraldehyde-3-phosphate dehydrogenase |
| Unigene8370_All | ns | ns | 11.69 | alcohol dehydrogenase (NADP^+^) |
| Unigene8294_All | ns | ns | 11.55 | glyceraldehyde 3-phosphate dehydrogenase |
| CL1203.Contig2_All | ns | ns | 11.24 | glyceraldehyde 3-phosphate dehydrogenase |
| CL1203.Contig1_All | ns | ns | 10.80 | glyceraldehyde 3-phosphate dehydrogenase |
| CL492.Contig2_All | ns | ns | 1.11 | glyceraldehyde 3-phosphate dehydrogenase |
| CL322.Contig1_All | 1.56 | ns | 1.66 | acetyl-CoA synthetase |
| Unigene669_All | ns | 11.30 | 10.96 | pyruvate dehydrogenase E2 component |
| **Starch and sucrose metabolism** | | | | |
| Unigene6875_All | 1.63 | ns | ns | glucokinase |
| Unigene97_All | 1.01 | ns | ns | phosphoglucomutase |
| CL780.Contig2_All | ns | 1.09 | ns | maltase-glucoamylase |
| Unigene4281_All | ns | -1.12 | ns | starch branching enzyme |
| Unigene8295_All | ns | ns | 11.75 | beta-glucosidase |
| Unigene8567_All | ns | ns | 11.69 | beta-glucosidase |
| CL742.Contig1_All | ns | 1.16 | 1.05 | UDP glucose 6-dehydrogenase |
| CL742.Contig2_All | 1.54 | 1.46 | 1.43 | UDP glucose 6-dehydrogenase |
| Unigene1125_All | -2.12 | -1.59 | -1.68 | maltase-glucoamylase |
| Unigene6325_All | 1.47 | 1.02 | 1.43 | isoamylase glycoside hydrolase family GH13 |
| **Pentose phosphate pathway** | | | | |
| CL240.Contig1_All | ns | 1.15 | ns | 6-phosphogluconate dehydrogenase |
| CL240.Contig2_All | ns | 1.52 | ns | 6-phosphogluconate dehydrogenase |
| CL240.Contig4_All | ns | 1.28 | ns | 6-phosphogluconate dehydrogenase |
| CL240.Contig5_All | ns | 1.39 | ns | 6-phosphogluconate dehydrogenase |
| CL240.Contig6_All | ns | 1.34 | ns | 6-phosphogluconate dehydrogenase |
| **Glycoside hydrolases (GH) and glycosyltransferases (GT)** | | | | |
| CL10.Contig2_All | 3.68 | ns | ns | alpha-glucosidase family GH31 |
| Unigene3893_All | 3.34 | ns | ns | alpha-glucosidase family GH31 |
| Unigene1729_All | 3.11 | ns | 2.43 | alpha-glucosidase family GH31 |
| Unigene2430_All | ns | 1.34 | ns | cellobiohydrolase A (1,4-beta-cellobiosidase A) |
| Unigene2742_All | ns | 1.07 | ns | CesA-like cellulose synthase (UDP-forming), family GT2 |
| CL150.Contig10_All | ns | 1.33 | ns | glycosyltransferase, family GT23 |
| CL150.Contig1_All | ns | 1.84 | 1.49 | glycosyltransferase, family GT23 |
| CL150.Contig12_All | ns | 1.84 | 1.66 | glycosyltransferase, family GT23 |
| CL150.Contig13_All | ns | 1.61 | 1.34 | glycosyltransferase, family GT23 |
| CL150.Contig14_All | ns | 1.71 | 1.40 | glycosyltransferase, family GT23 |
| CL150.Contig15_All | ns | 1.63 | 1.48 | glycosyltransferase, family GT23 |
| CL150.Contig16_All | ns | 1.70 | 1.60 | glycosyltransferase, family GT23 |
| CL150.Contig17_All | ns | 1.73 | 1.55 | glycosyltransferase, family GT23 |
| CL150.Contig18_All | ns | 1.63 | 1.47 | glycosyltransferase, family GT23 |
| CL150.Contig19_All | ns | 1.65 | 1.36 | glycosyltransferase, family GT23 |
| CL150.Contig2_All | ns | 1.88 | 1.64 | glycosyltransferase, family GT23 |
| CL150.Contig20_All | ns | 1.71 | 1.37 | glycosyltransferase, family GT23 |
| CL150.Contig21_All | ns | 1.80 | 1.49 | glycosyltransferase, family GT23 |
| CL150.Contig22_All | ns | 1.67 | 1.44 | glycosyltransferase, family GT23 |
| CL150.Contig23_All | ns | 1.60 | 1.46 | glycosyltransferase, family GT23 |
| CL150.Contig24_All | ns | 1.78 | 1.35 | glycosyltransferase, family GT23 |
| CL150.Contig25_All | ns | 1.81 | 1.61 | glycosyltransferase, family GT23 |
| CL150.Contig26_All | ns | 1.92 | 1.66 | glycosyltransferase, family GT23 |
| CL150.Contig27_All | ns | 1.88 | 1.52 | glycosyltransferase, family GT23 |
| CL150.Contig3_All | ns | 1.67 | 1.48 | glycosyltransferase, family GT23 |
| CL150.Contig4_All | ns | 1.76 | 1.59 | glycosyltransferase, family GT23 |
| CL150.Contig5_All | ns | 1.88 | 1.48 | glycosyltransferase, family GT23 |
| CL150.Contig6_All | ns | 1.63 | 1.46 | glycosyltransferase, family GT23 |
| CL150.Contig7_All | ns | 2.01 | 1.42 | glycosyltransferase, family GT23 |
| CL150.Contig8_All | ns | 1.61 | 1.36 | glycosyltransferase, family GT23 |
| Unigene2463_All | -2.18 | ns | -2.23 | glycoside hydrolase family GH16 endohydrolysis of (1-4)-beta-D-linkages of galactans |
| CL150.Contig9_All | 1.21 | 2.12 | 1.52 | glycosyltransferase, family GT23 |
| **(III) Protein synthesis-related DEGs** | | | | |
| **Ribosomal protein** | | | | |
| CL809.Contig2_All | 1.01 | ns | ns | 60S acidic ribosomal protein P0 |
| Unigene8167_All | ns | 4.23 | ns | 30S ribosomal protein S20 |
| Unigene6017_All | ns | 10.54 | ns | 60S ribosomal protein L10-1 |
| Unigene3689_All | ns | 1.85 | ns | 50S ribosomal protein L21 |
| CL567.Contig1_All | ns | 1.12 | ns | 50S ribosomal protein L17 |
| CL567.Contig4_All | ns | 1.26 | ns | 50S ribosomal protein L17 |
| Unigene1130_All | ns | 1.26 | ns | 50S ribosomal protein L3 |
| Unigene8165_All | ns | 2.80 | ns | 50S ribosomal protein L20 |
| Unigene2775_All | ns | -3.58 | ns | 40S ribosomal protein S10 |
| Unigene2980_All | ns | -3.00 | ns | 40S ribosomal protein S24-1 |
| Unigene3049_All | ns | -3.02 | ns | 40S ribosomal protein S25-3 |
| Unigene3305_All | ns | -2.59 | ns | 40S ribosomal protein S3-1 |
| Unigene3683_All | ns | -11.39 | ns | 40S ribosomal protein S16 |
| Unigene3696_All | ns | -3.21 | ns | 40S ribosomal protein S26 |
| Unigene4445_All | ns | -3.40 | ns | 40S ribosomal protein S28-1 |
| Unigene4495_All | ns | -4.52 | ns | 40S ribosomal protein S29 |
| Unigene5775_All | ns | -2.84 | ns | 40S ribosomal protein S23 |
| Unigene5972_All | ns | -4.40 | ns | 40S ribosomal protein S20-2 |
| Unigene653_All | ns | -3.64 | ns | 40S ribosomal protein S18 |
| Unigene6764_All | ns | -3.31 | ns | 40S ribosomal protein S15 |
| Unigene6884_All | ns | -1.74 | ns | ubiquitin-40S ribosomal protein S27a-2 |
| Unigene7634_All | ns | -2.74 | ns | 40S ribosomal protein S17-1 |
| CL1298.Contig2_All | ns | -1.59 | ns | 50S ribosomal protein L1 |
| Unigene6725_All | ns | -4.58 | ns | 60S ribosomal protein L27 |
| Unigene2691_All | ns | -3.26 | ns | 60S acidic ribosomal protein P0 |
| Unigene3022_All | ns | -3.52 | ns | 60S ribosomal protein L35a-2 |
| Unigene3684_All | ns | -3.26 | ns | 60S ribosomal protein L19-3 |
| Unigene1064_All | ns | -2.79 | ns | 60S ribosomal protein L12-3 |
| Unigene1235_All | ns | -2.52 | ns | 60S ribosomal protein L7-2 |
| Unigene1485_All | ns | -3.80 | ns | 60S ribosomal protein L21-1 |
| Unigene1575_All | ns | -3.74 | ns | 60S ribosomal protein L37-2 |
| Unigene1583_All | ns | -4.02 | ns | 60S ribosomal protein L34 |
| Unigene2045_All | ns | -3.36 | ns | 60S ribosomal protein L5 |
| Unigene2229_All | ns | -3.94 | ns | 60S ribosomal protein L38 |
| Unigene431_All | ns | -2.36 | ns | 60S acidic ribosomal protein P1-2 |
| Unigene4726_All | ns | -2.32 | ns | 60S ribosomal protein L4-1 |
| Unigene4862_All | ns | -3.33 | ns | 60S ribosomal protein L27a-3 |
| Unigene5193_All | ns | -3.64 | ns | 60S ribosomal protein L17 |
| Unigene5198_All | ns | -2.57 | ns | 60S ribosomal protein L22-2 |
| Unigene5231_All | ns | -2.69 | ns | 60S ribosomal protein L31 |
| Unigene5237_All | ns | -2.98 | ns | 60S ribosomal protein L32-1 |
| Unigene5238_All | ns | -2.52 | ns | 60S ribosomal protein L8-1 |
| Unigene5278_All | ns | -11.16 | ns | 60S ribosomal protein L37a |
| Unigene5678_All | ns | -4.98 | ns | 60S ribosomal protein L11-1 |
| Unigene5996_All | ns | -4.52 | ns | 60S ribosomal protein L36-3 |
| Unigene5997_All | ns | -3.26 | ns | 60S ribosomal protein L13-1 |
| Unigene6411_All | ns | -12.33 | ns | 60S acidic ribosomal protein P2-2 |
| Unigene6962_All | ns | -2.69 | ns | 60S ribosomal protein L10-1 |
| Unigene7483_All | ns | -3.16 | ns | 60S ribosomal protein L30-2 |
| Unigene7595_All | ns | -3.46 | ns | 60S ribosomal protein L3 |
| Unigene8525_All | ns | ns | 11.43 | 40S ribosomal protein S8 |
| Unigene8627_All | ns | ns | 11.53 | 40S ribosomal protein S27-2 |
| CL858.Contig1_All | ns | ns | 11.02 | 60S ribosomal protein L38 |
| Unigene8323_All | ns | ns | 11.87 | 60S ribosomal protein L41 |
| Unigene8325_All | ns | ns | 11.19 | 60S ribosomal protein L32-1 |
| Unigene8358_All | ns | ns | 11.37 | 60S acidic ribosomal protein P2B |
| Unigene8439_All | ns | ns | 11.18 | 60S ribosomal protein L37a |
| Unigene8450_All | ns | ns | 10.98 | 60S acidic ribosomal protein P1 |
| Unigene8457_All | ns | ns | 10.93 | 60S ribosomal protein L3 |
| Unigene8638_All | ns | ns | 11.25 | 60S ribosomal protein L3-2 |
| Unigene8642_All | ns | ns | 11.10 | 60S ribosomal protein L30 |
| Unigene8785_All | ns | ns | 10.60 | 60S ribosomal protein L18-2 |
| CL824.Contig1_All | 2.72 | ns | 2.08 | ribosomal protein S10 |
| Unigene5296_All | -2.23 | -2.84 | ns | 40S ribosomal protein S8-2 |
| Unigene5973_All | -2.58 | -3.35 | ns | 40S ribosomal protein S12 |
| Unigene6468_All | -2.01 | -4.36 | ns | 40S ribosomal protein S9-1 |
| Unigene1272_All | -1.94 | -3.56 | ns | 60S ribosomal protein L26-1 |
| Unigene2233_All | -2.90 | -3.52 | ns | 60S ribosomal protein L3 |
| Unigene6021_All | -2.11 | -3.46 | ns | 60S ribosomal protein L18-3 |
| Unigene6513_All | -1.98 | -2.18 | ns | 60S ribosomal protein L9 |
| Unigene7206_All | -1.83 | -2.86 | ns | 60S ribosomal protein L13a |
| Unigene7577_All | -2.39 | -4.74 | ns | 60S ribosomal protein L6 |
| Unigene3737_All | -2.66 | -3.69 | ns | 60S ribosomal protein L23a |
| Unigene5739_All | ns | -3.42 | -1.41 | 40S ribosomal protein S3a |
| Unigene4423_All | ns | -4.22 | -2.40 | 40S ribosomal protein S13 |
| Unigene4431_All | ns | -3.98 | -2.65 | 40S ribosomal protein S5 |
| Unigene1709_All | ns | -3.08 | -1.29 | 40S ribosomal protein S2-2 |
| Unigene2324_All | ns | -3.26 | -3.24 | 40S ribosomal protein S21 |
| Unigene5266_All | ns | -2.88 | -3.45 | 60S ribosomal protein L39 |
| Unigene7507_All | ns | -3.33 | -11.34 | 60S ribosomal protein L41 |
| Unigene7557_All | ns | -4.11 | -4.09 | 60S acidic ribosomal protein P0 |
| Unigene1163_All | ns | -2.59 | -1.68 | 60S ribosomal protein L7a-1 |
| Unigene4302_All | -1.84 | -3.88 | -1.54 | 60S ribosomal protein L15-2 |
| Unigene4464_All | -2.46 | -2.91 | -1.67 | 60S ribosomal protein L10a-2 |
| Unigene7628_All | -2.81 | -3.26 | -2.24 | 60S ribosomal protein L23 |
| Unigene1079_All | -1.61 | -3.17 | -1.83 | 40S ribosomal protein S6 |
| Unigene2321_All | -2.05 | -2.31 | -2.29 | 40S ribosomal protein S15a |
| Unigene4484_All | -2.32 | -2.62 | -2.12 | 40S ribosomal protein S4 |
| **Translation-related protein** | | | | |
| Unigene4246_All | ns | -1.11 | ns | tyrosine--tRNA ligase |
| Unigene3285_All | ns | -1.86 | ns | translation initiation factor IF-2 |
| Unigene997_All | ns | -2.43 | ns | elongation translation factor 1 alpha |
| CL1496.Contig1_All | ns | 1.60 | ns | lysine-tRNA ligase |
| Unigene3576_All | ns | 1.08 | ns | lysine-tRNA ligase |
| Unigene5129_All | ns | 1.04 | ns | tryptophan--tRNA ligase |
| CL523.Contig2_All | ns | 1.14 | ns | leucyl-tRNA synthetase |
| Unigene5448_All | ns | 1.07 | ns | valine--tRNA ligase |
| Unigene475_All | ns | 1.10 | ns | asparagine--tRNA ligase |
| Unigene3487_All | ns | 2.10 | ns | translation initiation factor IF-3 |
| Unigene6883_All | ns | 1.67 | ns | translation initiation factor eIF2B |
| CL1474.Contig4_All | ns | 1.24 | ns | translation initiation factor eIF2B |
| CL1474.Contig2_All | ns | 1.08 | ns | translation initiation factor eIF2B |
| Unigene2408_All | ns | 1.02 | ns | translation initiation factor 4A |
| Unigene3839_All | ns | 1.81 | ns | elongation factor Tu |
| Unigene8436_All | ns | ns | 11.42 | translation initiation factor 5B |
| CL901.Contig1_All | ns | ns | 1.79 | translation initiation factor IF-2 |
| Unigene8301_All | ns | ns | 10.91 | translation initiation factor 4A |
| Unigene8313_All | ns | ns | 10.64 | translation initiation factor 4A |
| Unigene8542_All | ns | ns | 11.50 | translation initiation factor 5A |
| Unigene8505_All | ns | ns | 12.02 | translation initiation factor eIF-1 |
| Unigene2313_All | ns | ns | 10.87 | translation initiation factor IF-2 |
| CL879.Contig1_All | ns | ns | 12.17 | elongation factor 1-alpha |
| Unigene8571_All | ns | ns | 11.68 | elongation factor 1-alpha |
| CL103.Contig10_All | ns | ns | 1.48 | translation elongation factors (GTPases) |
| CL103.Contig12_All | ns | ns | 1.64 | translation elongation factors (GTPases) |
| CL103.Contig13_All | ns | ns | 1.49 | translation elongation factors (GTPases) |
| CL103.Contig14_All | ns | ns | 1.56 | translation elongation factors (GTPases) |
| CL103.Contig15_All | ns | ns | 1.40 | translation elongation factors (GTPases) |
| CL103.Contig16_All | ns | ns | 1.64 | translation elongation factors (GTPases) |
| CL103.Contig17_All | ns | ns | 1.49 | translation elongation factors (GTPases) |
| CL103.Contig18_All | ns | ns | 1.56 | translation elongation factors (GTPases) |
| CL103.Contig19_All | ns | ns | 1.58 | translation elongation factors (GTPases) |
| CL103.Contig2_All | ns | ns | 1.49 | translation elongation factors (GTPases) |
| CL103.Contig20_All | ns | ns | 1.40 | translation elongation factors (GTPases) |
| CL103.Contig21_All | ns | ns | 1.58 | translation elongation factors (GTPases) |
| CL103.Contig22_All | ns | ns | 1.56 | translation elongation factors (GTPases) |
| CL103.Contig23_All | ns | ns | 1.63 | translation elongation factors (GTPases) |
| CL103.Contig25_All | ns | ns | 1.40 | translation elongation factors (GTPases) |
| CL103.Contig3_All | ns | ns | 1.56 | translation elongation factors (GTPases) |
| CL103.Contig4_All | ns | ns | 1.49 | translation elongation factors (GTPases) |
| CL103.Contig44_All | ns | ns | 2.56 | translation elongation factors (GTPases) |
| CL103.Contig45_All | ns | ns | 1.45 | translation elongation factors (GTPases) |
| CL103.Contig46_All | ns | ns | 1.86 | translation elongation factors (GTPases) |
| CL103.Contig49_All | ns | ns | 1.86 | translation elongation factors (GTPases) |
| CL103.Contig5_All | ns | ns | 1.60 | translation elongation factors (GTPases) |
| CL103.Contig50_All | ns | ns | 1.76 | translation elongation factors (GTPases) |
| CL103.Contig51_All | ns | ns | 1.49 | translation elongation factors (GTPases) |
| CL103.Contig53_All | ns | ns | 1.64 | translation elongation factors (GTPases) |
| CL103.Contig54_All | ns | ns | 1.54 | translation elongation factors (GTPases) |
| CL103.Contig55_All | ns | ns | 1.58 | translation elongation factors (GTPases) |
| CL103.Contig56_All | ns | ns | 1.58 | translation elongation factors (GTPases) |
| CL103.Contig58_All | ns | ns | 1.56 | translation elongation factors (GTPases) |
| CL103.Contig60_All | ns | ns | 1.86 | translation elongation factors (GTPases) |
| CL103.Contig62_All | ns | ns | 1.86 | translation elongation factors (GTPases) |
| CL103.Contig64_All | ns | ns | 1.54 | translation elongation factors (GTPases) |
| CL103.Contig65_All | ns | ns | 1.54 | translation elongation factors (GTPases) |
| CL103.Contig66_All | ns | ns | 1.33 | translation elongation factors (GTPases) |
| CL103.Contig67_All | ns | ns | 1.56 | translation elongation factors (GTPases) |
| CL103.Contig68_All | ns | ns | 1.14 | translation elongation factors (GTPases) |
| CL103.Contig71_All | ns | ns | 1.64 | translation elongation factors (GTPases) |
| CL103.Contig72_All | ns | ns | 1.54 | translation elongation factors (GTPases) |
| CL103.Contig8_All | ns | ns | 1.64 | translation elongation factors (GTPases) |
| Unigene5899_All | -2.35 | ns | -1.71 | translation initiation inhibitor, yjgF family |
| Unigene4835_All | ns | 10.65 | 10.98 | translation initiation factor IF-2 |
| Unigene2542_All | ns | 11.41 | 11.58 | elongation factor 1-alpha |
| Unigene7590_All | -1.97 | -2.88 | -2.64 | translation elongation factor eEF1 |
| **(IV) Heat shock-related DEGs** | | | | |
| **Hsp100 family** | | | | |
| Unigene1296_All | ns | -1.40 | ns | ATP-dependent Clp protease ATP-binding subunit ClpB |
| Unigene8444_All | ns | ns | 11.05 | ATP-dependent Clp protease ATP-binding subunit ClpB |
| Unigene8616_All | ns | ns | 11.28 | ATP-dependent Clp protease ATP-binding subunit ClpB |
| CL835.Contig6_All | ns | ns | 1.51 | ATP-dependent Clp protease adaptor protein ClpS |
| Unigene8482_All | ns | ns | 11.66 | ATP-dependent Clp protease ATP-binding subunit ClpB |
| **Hsp90 family** | | | | |
| Unigene8636_All | ns | ns | 10.87 | heat shock protein 90kDa beta |
| CL1461.Contig2_All | ns | ns | 11.25 | heat shock protein 82 |
| Unigene8300_All | ns | ns | 11.22 | heat shock protein 83 |
| Unigene8524_All | ns | ns | 11.79 | heat shock protein 90kDa beta |
| CL660.Contig3_All | ns | ns | 11.68 | heat shock protein 83 |
| CL1461.Contig1_All | ns | ns | 11.38 | heat shock protein 82 |
| CL660.Contig2_All | ns | ns | 12.15 | heat shock cognate protein 80 |
| Unigene8522_All | ns | ns | 11.73 | heat shock protein 90kDa beta |
| **Hsp70 family** | | | | |
| Unigene2813_All | ns | -1.27 | ns | heat shock 70kDa protein 4 |
| CL335.Contig1_All | ns | -1.11 | ns | heat shock 70kDa protein 1/8 |
| Unigene7650_All | ns | -4.58 | ns | heat shock 70kDa protein 5 |
| Unigene7650_All | ns | ns | -2.57 | luminal-binding protein 2 |
| Unigene8614_All | ns | ns | 11.59 | luminal-binding protein 5 |
| CL576.Contig1_All | ns | ns | 11.59 | heat shock 70 kDa protein |
| Unigene8544_All | ns | ns | 11.54 | heat shock 70 kDa protein 5 |
| Unigene8556_All | ns | ns | 11.53 | luminal-binding protein 4 |
| Unigene8311_All | ns | ns | 11.48 | luminal-binding protein 3 |
| Unigene8918_All | ns | ns | 10.58 | heat shock cognate 70 kDa protein |
| CL822.Contig1_All | ns | ns | 11.37 | heat shock 70kDa protein |
| CL1488.Contig1_All | ns | ns | 11.23 | heat shock cognate 70 kDa protein |
| Unigene8393_All | ns | ns | 11.03 | heat shock protein 70 |
| Unigene8322_All | ns | ns | 10.96 | luminal-binding protein 5 |
| Unigene8684_All | ns | ns | 10.91 | heat shock 70 kDa protein |
| CL822.Contig2_All | ns | ns | 6.08 | heat shock protein 70 cognate |
| **DnaJ homolog subfamily** | | | | |
| CL919.Contig1_All | ns | -1.11 | ns | DnaJ protein homolog ANJ1 |
| CL919.Contig2_All | ns | -2.08 | ns | DnaJ protein homolog ANJ1 |
| CL1139.Contig2_All | ns | -1.51 | ns | DnaJ protein homolog 2 |
| CL430.Contig2_All | ns | -1.09 | ns | chaperone protein dnaJ 1, mitochondrial |
| Unigene3220_All | ns | 1.26 | ns | chaperone protein dnaJ 15 |
| Unigene8396_All | ns | ns | 11.38 | chaperone protein dnaJ 8 |
| CL1174.Contig2_All | ns | ns | 11.73 | DnaJ protein homolog |
| Unigene8609_All | ns | ns | 11.45 | chaperone protein dnaJ 72 |
| CL845.Contig1_All | ns | ns | 10.90 | protein DJ-1 homolog D |
| **Hsp60 family** | | | | |
| Unigene2503_All | ns | 2.97 | ns | chaperonin GroEL |
| Unigene8540_All | ns | ns | 11.16 | chaperonin 60 subunit beta 2 |
| Unigene8471_All | ns | ns | 11.04 | chaperonin CPN60-2 |
| Unigene8350_All | ns | ns | 11.33 | chaperonin GroEL |
| Unigene8456_All | ns | ns | 11.08 | chaperonin GroEL |
| Unigene8472_All | ns | ns | 11.34 | chaperonin GroEL |
| **Small Hsp** | | | | |
| CL1028.Contig2_All | ns | -1.40 | ns | co-chaperonin GroES (Hsp10) |
| CL635.Contig1_All | ns | ns | 13.92 | 18.5 kDa class I heat shock protein |
| CL1487.Contig1_All | ns | ns | 13.48 | 17.3 kDa class I heat shock protein |
| Unigene8583_All | ns | ns | 13.47 | 17.3 kDa class II heat shock protein |
| Unigene8582_All | ns | ns | 13.16 | 18.5 kDa class I heat shock protein |
| Unigene8575_All | ns | ns | 12.80 | 23.6 kDa heat shock protein |
| Unigene8550_All | ns | ns | 12.65 | 17.4 kDa class I heat shock protein |
| CL635.Contig2_All | ns | ns | 12.24 | 18.5 kDa class I heat shock protein |
| Unigene8586_All | ns | ns | 12.17 | 17.3 kDa class I heat shock protein |
| Unigene8546_All | ns | ns | 11.63 | 17.4 kDa class III heat shock protein |
| Unigene8551_All | ns | ns | 12.14 | 17.8 kDa class I heat shock protein |
| Unigene8287_All | ns | ns | 11.86 | 22.0 kDa class IV heat shock protein |
| Unigene8559_All | ns | ns | 11.82 | heat shock 22K family protein |
| Unigene8600_All | ns | ns | 11.29 | 15.7 kDa heat shock protein |
| CL628.Contig1_All | ns | ns | 10.86 | 22.0 kDa class IV heat shock protein |
| Unigene8628_All | ns | ns | 11.16 | small heat shock protein |
| Unigene8630_All | ns | ns | 11.07 | small heat shock protein |
| **(V) ROS-related DEGs** | | | | |
| CL402.Contig2_All | 1.56 | ns | ns | vanadium-dependent bromoperoxidase 1 |
| CL402.Contig5_All | 1.37 | ns | ns | vanadium-dependent bromoperoxidase 1 |
| CL402.Contig1_All | 1.24 | ns | ns | vanadium-dependent bromoperoxidase 1 |
| CL402.Contig4_All | 1.15 | ns | ns | vanadium-dependent bromoperoxidase 1 |
| CL1021.Contig1_All | ns | -1.64 | ns | glutathione S-transferase |
| Unigene2196_All | ns | 2.39 | ns | L-ascorbate peroxidase |
| CL1225.Contig1_All | ns | 1.07 | ns | L-ascorbate peroxidase |
| CL1225.Contig2_All | ns | 1.01 | ns | L-ascorbate peroxidase |
| Unigene2760_All | ns | 1.47 | ns | glutathione S-transferase |
| Unigene455_All | ns | 1.36 | ns | NADPH oxidase, respiratory burst oxidase homologue |
| CL773.Contig2_All | ns | ns | 1.01 | peroxiredoxin 6, 1-Cys peroxiredoxin |
| CL773.Contig1_All | ns | ns | 1.04 | peroxiredoxin 6, 1-Cys peroxiredoxin |
| CL151.Contig1_All | ns | ns | 1.04 | L-ascorbate peroxidase |
| CL849.Contig2_All | ns | ns | 11.02 | L-ascorbate peroxidase |
| CL849.Contig1_All | ns | ns | 10.62 | L-ascorbate peroxidase |
| CL849.Contig3_All | ns | ns | 11.62 | L-ascorbate peroxidase |
| Unigene8510_All | ns | ns | 11.28 | catalase CAT1 |
| CL1210.Contig1_All | ns | ns | 11.67 | catalase CAT2 |
| Unigene8316_All | ns | ns | 11.18 | glutathione S-transferase |
| Unigene8610_All | ns | ns | 11.32 | glutathione S-transferase |
| Unigene8587_All | ns | ns | 12.40 | peroxiredoxin (alkyl hydroperoxide reductase subunit C) |
| Unigene8599_All | ns | ns | 11.59 | glutaredoxin |
| Unigene8557_All | ns | ns | 11.91 | Cu/Zn superoxide dismutase |
| Unigene8607_All | ns | ns | 11.43 | peroxidase |
| Unigene8626_All | ns | ns | 11.35 | peroxidase |
| Unigene8596_All | ns | ns | 11.66 | peroxidase |
| Unigene8541_All | ns | ns | 10.92 | thioredoxin H-type 3 |
| Unigene8601_All | ns | ns | 11.35 | DHAR class glutathione transferase DHAR2 |
| Unigene4664_All | 1.76 | ns | 1.70 | respiratory burst oxidase homolog |
| Unigene5161_All | 1.54 | ns | 1.72 | catalase |
| Unigene2550_All | 1.96 | ns | 1.25 | glutathione S-transferase |
| Unigene3114_All | 2.37 | ns | 2.20 | methionine sulfoxide reductase 1 |
| Unigene940_All | 4.32 | 4.10 | 2.22 | respiratory burst oxidase homolog |
| Unigene107_All | 1.04 | 1.11 | 1.04 | glutathione S-transferase |
| Unigene5138_All | 1.71 | 2.70 | 1.32 | peroxiredoxin Q/BCP |
| **(VI) Calcium signaling-associated DEGs** | | | | |
| Unigene3650_All | ns | -1.10 | ns | Ca^2+^/calmodulin-dependent protein kinase |
| Unigene1314_All | ns | 1.60 | ns | CBL-interacting serine/threonine-protein kinase 6-like |
| Unigene4206_All | ns | 1.20 | ns | CBL-interacting serine/threonine-protein kinase 3 |
| Unigene7390_All | ns | 1.09 | ns | calcium-binding EF hand family protein |
| Unigene8320_All | ns | ns | 11.29 | CBL-interacting serine/threonine-protein kinase |
| CL617.Contig3_All | ns | ns | 1.01 | CBL-interacting protein kinase 23 |
| Unigene6144_All | ns | ns | 1.01 | Ca^2+^/calmodulin-dependent protein kinase |
| Unigene8585_All | ns | ns | 12.55 | calreticulin |
| Unigene8597_All | ns | ns | 11.60 | calmodulin |
| Unigene8547_All | ns | ns | 11.28 | calmodulin |
| Unigene8339_All | ns | ns | 11.36 | calnexin |
| Unigene8737_All | ns | ns | 10.82 | calcium ion binding protein |
| Unigene8751_All | ns | ns | 10.81 | calcyclin-binding protein |
| Unigene6249_All | 2.07 | ns | 2.08 | calcium-dependent protein kinase |
| Unigene7636_All | -2.49 | -3.52 | -3.09 | calreticulin |
| **(VII) Phytohormone-associated DEGs** | | | | |
| Unigene5660_All | -1.39 | ns | ns | serine/threonine-protein kinase CTR1-like |
| Unigene2340_All | -4.15 | ns | ns | adenylate isopentenyltransferase (cytokinin synthase) |
| CL1424.Contig2_All | ns | 1.27 | ns | auxin efflux carrier |
| CL1424.Contig1_All | ns | 2.80 | ns | auxin efflux carrier |
| Unigene544_All | ns | 1.84 | ns | serine/threonine-protein kinase CTR1 |
| Unigene7283_All | ns | 1.01 | ns | serine/threonine-protein kinase CTR1 |
| Unigene2561_All | ns | 2.06 | ns | ompR transcriptional regulator (chloroplast) |
| Unigene309_All | ns | -2.26 | ns | two-component response regulator ARR-B family |
| Unigene6577_All | ns | -1.01 | ns | SNF1-related protein kinase regulatory subunit beta-2 |
| Unigene8855_All | ns | ns | 10.94 | auxin-responsive protein IAA9 |
| Unigene8564_All | ns | ns | 13.63 | auxin-repressed protein-like protein ARP1 |
| Unigene8658_All | ns | ns | 11.08 | ethylene-insensitive protein 3 |
| Unigene8671_All | ns | ns | 11.09 | ethylene-insensitive protein 3 |
| CL1283.Contig1_All | ns | ns | -1.51 | spermine oxidase |
| CL527.Contig1_All | ns | ns | 1.63 | adenylate isopentenyltransferase (cytokinin synthase) |
| Unigene8591_All | ns | ns | 11.43 | brassinosteroid insensitive 1 |
| Unigene8345_All | ns | ns | 11.60 | arabidopsis histidine kinase 2/3/4 (cytokinin receptor) |
| Unigene8708_All | ns | ns | 10.96 | GAST1 protein precursor |
| Unigene2715_All | 1.06 | 1.79 | 1.70 | abscisic acid insensitive 4 (ABI4) |

All of these genes with FDR cut off of less than 0.001. Positive and negative values indicate genes were up- and down-regulated by SA or/and MJ, respectively. ‘ns’ represents no significant difference .
