# Supplementary material for: Comparative transcriptional profiling of Gracilariopsis lemaneiformis in response to salicylic acid- and methyl jasmonate-mediated heat resistance
Source: PLoS One. 2017 May 2;12(5):e0176531. doi: 10.1371/journal.pone.0176531 (PMC5413009; doi:10.1371/journal.pone.0176531)
Supplement: S8 Table — (DOC) [file pone.0176531.s010.doc]

S8 Table. Comparison of the DEG expression pattern between RNA-seq and qPCR analysis

| Gene ID | Gene annotation | Treatment | RNA-seq analysis | | qPCR analysis | |
| --- | --- | --- | --- | --- | --- | --- |
|  |  |  | Fold change | Up/Down | Fold change | Up/Down |
| **Class I** | | | | | | |
| Unigene1125_All | alpha-1,4-glucan lyase isozyme | SA | 0.23 | Down* | 0.45 | Down* |
| Unigene4281_All | starch-branching enzyme | MJ | 0.46 | Down* | 0.72 | Down* |
| Unigene5895_All | zeta-carotene desaturase | MJ | 0.36 | Down* | 0.57 | Down* |
| CL1163.Contig1_All | haloalkane dehalogenase | SA | 1.65 | Up* | 2.54 | Up* |
| CL1163.Contig1_All | haloalkane dehalogenase | MJ | 2.53 | Up* | 3.85 | Up* |
| Unigene4281_All | starch-branching enzyme | SA | 1.11 | Up | 1.67 | Up* |
| CL240.Contig1_All | 6-phosphogluconate dehydrogenase | SA/MJ | 1.58 | Up | 2.37 | Up* |
| Unigene2767_All | alanine-glyoxylate aminotransferase | SA | 1.13 | Up | 1.61 | Up* |
| CL402.Contig1_All | vanadium-dependent bromoperoxidase 1 | MJ | 1.55 | Up | 2.17 | Up* |
| Unigene2196_All | ascorbate peroxidase | SA/MJ | 1.96 | Up* | 2.63 | Up* |
| Unigene455_All | NADPH oxidase | MJ | 2.57 | Up* | 3.33 | Up* |
| CL1283.Contig1_All | spermine oxidase | SA | 0.52 | Down* | 0.65 | Down* |
| CL1424.Contig1_All | auxin efflux carrier | SA/MJ | 1.41 | Up | 1.74 | Up* |
| Unigene5895_All | zeta-carotene desaturase | SA | 1.48 | Up* | 1.74 | Up* |
| CL402.Contig1_All | vanadium-dependent bromoperoxidase 1 | SA/MJ | 1.93 | Up | 2.22 | Up* |
| Unigene7277_All | glyoxylate reductase | SA | 1.20 | Up* | 1.37 | Up* |
| Unigene1125_All | alpha-1,4-glucan lyase isozyme | SA/MJ | 0.31 | Down* | 0.34 | Down* |
| CL335.Contig1_All | heat shock protein 70 | SA | 0.77 | Down* | 0.83 | Down* |
| CL335.Contig1_All | heat shock protein 70 | SA/MJ | 0.79 | Down* | 0.85 | Down* |
| Unigene455_All | NADPH oxidase | SA | 0.75 | Down* | 0.79 | Down |
| CL1175.Contig1_All | fructose-1,6-biphosphate aldolase | SA/MJ | 0.94 | Down | 0.99 | Down |
| CL1486.Contig2_All | pyruvate kinase | SA/MJ | 1.42 | Up | 1.47 | Up* |
| CL402.Contig1_All | vanadium-dependent bromoperoxidase 1 | SA | 2.36 | Up* | 2.44 | Up* |
| Unigene5267_All | acetyl-CoA carboxylase carboxytransferase | SA | 0.80 | Down | 0.83 | Down |
| Unigene2126_All | 1-acyl-sn-glycerol-3-phosphate acyltransferase | SA | 1.02 | Up | 1.02 | Up |
| Unigene1125_All | alpha-1,4-glucan lyase isozyme | MJ | 0.33 | Down* | 0.33 | Down* |
| Unigene567_All | omega-3 fatty acid desaturase | SA/MJ | 2.14 | Up* | 2.09 | Up* |
| Unigene567_All | omega-3 fatty acid desaturase | MJ | 1.38 | Up | 1.35 | Up* |
| CL492.Contig3_All | glyceraldehyde-3-phosphate dehydrogenase | SA/MJ | 1.85 | Up* | 1.80 | Up* |
| Unigene2550_All | glutathione S-transferase | MJ | 1.75 | Up* | 1.69 | Up* |
| Unigene2126_All | 1-acyl-sn-glycerol-3-phosphate acyltransferase | SA/MJ | 0.97 | Down | 0.90 | Down |
| CL1283.Contig1_All | spermine oxidase | MJ | 0.82 | Down* | 0.73 | Down* |
| Unigene6851_All | photosystem II CP47 chlorophyll apoprotein | SA | 1.62 | Up | 1.44 | Up* |
| Unigene93_All | phycobilisome linker polypeptide | SA | 1.63 | Up | 1.44 | Up* |
| CL240.Contig1_All | 6-phosphogluconate dehydrogenase, | MJ | 2.22 | Up* | 1.93 | Up* |
| Unigene2550_All | glutathione S-transferase | SM | 2.37 | Up* | 2.03 | Up* |
| Unigene7277_All | glyoxylate reductase | SA/MJ | 1.47 | Up* | 1.25 | Up* |
| Unigene3077_All | light-harvesting protein | MJ | 1.45 | Up | 1.19 | Up* |
| Unigene3077_All | light-harvesting protein | SA/MJ | 1.76 | Up* | 1.41 | Up* |
| CL492.Contig3_All | glyceraldehyde-3-phosphate dehydrogenase | MJ | 1.59 | Up | 1.25 | Up* |
| CL1486.Contig2_All | pyruvate kinase | SA | 1.51 | Up | 1.17 | Up |
| Unigene2196_All | ascorbate peroxidase | SA | 1.94 | Up* | 1.44 | Up* |
| Unigene5895_All | zeta-carotene desaturase | SA/MJ | 1.64 | Up* | 1.17 | Up |
| CL1424.Contig1_All | auxin efflux carrier | SA | 2.49546 | Up | 1.77 | Up* |
| Unigene5514_All | cytochrome b6 | SA | 2.01 | Up | 1.37 | Up* |
| Unigene5649_All | phycoerythrin beta subunit | SA | 2.12 | Up* | 1.36 | Up* |
| CL240.Contig1_All | 6-phosphogluconate dehydrogenase, | SA | 1.82 | Up | 1.17 | Up |
| Unigene3077_All | light-harvesting protein | SA | 2.09 | Up* | 1.32 | Up* |
| Unigene4373_All | proline-rich receptor-like protein kinase PERK1 | SA | 2.36 | Up* | 1.50 | Up* |
| Unigene567_All | omega-3 fatty acid desaturase | SA | 2.23 | Up* | 1.40 | Up* |
| Unigene5649_All | phycoerythrin beta subunit | MJ | 1.83 | Up* | 1.14 | Up |
| Unigene6851_All | photosystem II CP47 chlorophyll apoprotein | SA/MJ | 2.02 | Up | 1.24 | Up* |
| CL1486.Contig2_All | pyruvate kinase | MJ | 2.20 | Up* | 1.32 | Up* |
| Unigene2767_All | alanine-glyoxylate aminotransferase | SA/MJ | 0.84 | Down* | 0.49 | Down* |
| CL492.Contig3_All | glyceraldehyde -3-phosphate dehydrogenase | SA | 2.16 | Up* | 1.23 | Up* |
| Unigene2196_All | ascorbate peroxidase | MJ | 5.23 | Up* | 2.98 | Up* |
| Unigene2550_All | glutathione S-transferase | SA | 3.89 | Up* | 2.11 | Up* |
| Unigene4373_All | proline-rich receptor-like protein kinase PERK1 | SA/MJ | 2.51 | Up* | 1.31 | Up* |
| Unigene5514_All | cytochrome b6 | SA/MJ | 2.49 | Up* | 1.26 | Up* |
| **Class II** | | | | | | |
| Unigene2767_All | alanine-glyoxylate aminotransferase | MJ | 0.10 | Down* | 0.52 | Down* |
| Unigene2126_All | 1-acyl-sn-glycerol-3-phosphate acyltransferase | MJ | 0.16 | Down* | 0.83 | Down* |
| Unigene2463_All | glycoside hydrolase family GH16 endohydrolysis of (1-4)-beta-D-linkages of galactans | SA/MJ | 0.21 | Down* | 0.94 | Down |
| CL1175.Contig1_All | fructose-1,6-biphosphate aldolase | MJ | 0.16 | Down* | 0.70 | Down* |
| Unigene2463_All | glycoside hydrolase family GH16 endohydrolysis of (1-4)-beta-D-linkages of galactans | SA | 0.22 | Down* | 0.92 | Down |
| Unigene7277_All | glyoxylate reductase | MJ | 0.28 | Down* | 0.78 | Down* |
| Unigene5267_All | acetyl-CoA carboxylase carboxytransferase | SA/MJ | 1.05 | Up | 2.94 | Up* |
| Unigene5660_All | serine/threonine -protein kinase CTR1-like | SA | 0.38 | Down* | 0.95 | Down |
| CL1283.Contig1_All | spermine oxidase | SA/MJ | 0.35 | Down* | 0.84 | Down* |
| CL1163.Contig1_All | haloalkane dehalogenase | SA/MJ | 1.20 | Up* | 2.56 | Up* |
| Unigene121_All | 3-oxoacyl-acyl-carrier-protein synthase 3 | SA/MJ | 2.58 | Up | 1.18 | Up* |
| Unigene93_All | phycobilisome linker polypeptide | SA/MJ | 2.80 | Up* | 1.18 | Up* |
| Unigene5649_All | phycoerythrin beta subunit | SA/MJ | 2.44 | Up* | 1.02 | Up |
| Unigene121_All | 3-oxoacyl-acyl-carrier-protein synthase 3 | MJ | 3.54 | Up* | 1.47 | Up* |
| Unigene56_All | ribulose-1,5-bisphosphate carboxylase/oxygenase large subunit | SA | 3.35 | Up* | 1.21 | Up* |
| Unigene56_All | ribulose-1,5-bisphosphate carboxylase/oxygenase large subunit | SA/MJ | 3.08 | Up* | 1.01 | Up |
| Unigene5514_All | cytochrome b6 | MJ | 4.43 | Up* | 1.43663 | Up* |
| Unigene5267_All | acetyl-CoA carboxylase carboxytransferase | MJ | 8.62 | Up* | 2.77 | Up* |
| CL1424.Contig1_All | auxin efflux carrier | MJ | 6.97 | Up* | 1.92 | Up* |
| Unigene6851_All | photosystem II CP47 chlorophyll apoprotein | MJ | 5.73 | Up* | 1.37 | Up* |
| Unigene93_All | phycobilisome linker polypeptide | MJ | 6.95 | Up* | 1.03 | Up |
| Unigene56_All | ribulose-1,5-bisphosphate carboxylase/oxygenase large subunit | MJ | 13.85 | Up* | 1.45 | Up* |
| **Class III** | | | | | | |
| CL335.Contig1_All | heat shock protein 70 | MJ | 0.46 | Down* | 1.41 | Up* |
| Unigene5660_All | serine/threonine -protein kinase CTR1-like | MJ | 0.73 | Down | 4.59 | Up* |
| Unigene5660_All | serine/threonine -protein kinase CTR1-like | SA/MJ | 0.53 | Down* | 6.48 | Up* |
| Unigene455_All | NADPH oxidase | SA/MJ | 0.77 | Down* | 2.05 | Up* |
| Unigene4281_All | starch-branching enzyme | SA/MJ | 1.16 | Up* | 0.66 | Down* |
| Unigene4373_All | proline-rich receptor-like protein kinase PERK1 | MJ | 1.72 | Up* | 0.62 | Down* |
| Unigene2463_All | glycoside hydrolase family GH16 endohydrolysis of (1-4)-beta-D-linkages of galactans | MJ | 1.06 | Up | 0.66 | Down* |
| CL1175.Contig1_All | fructose-1,6-biphosphate aldolase | SA | 0.82 | Down | 1.04 | Up* |
| Unigene121_All | 3-oxoacyl-acyl-carrier-protein synthase 3 | SA | 0.95 | Down | 1.45 | Up* |

‘*’ in the RNA-seq data represents FDR < 0.001. ‘*’ in the qPCR data represents *P* < 0.05. Positive and negative values indicate genes were up- and down-regulated, respectively.
